# Metabolic dysfunction, antidiabetic drugs, and asthma: bibliometric evidence, emerging trends, and translational frontiers from 2006 to 2025

**DOI:** 10.3389/fendo.2026.1883559

**Published:** 2026-07-22

**Authors:** Rong Jiang, Jie Wang, Siping Wang

**Affiliations:** 1 Department of Nursing, Yantaishan Hospital, Yantai, Shandong, China; 2 Department of Trauma Surgery I, Yantaishan Hospital, Yantai, Shandong, China; 3 Department of Gastroenterology I, Yantaishan Hospital, Yantai, Shandong, China

**Keywords:** antidiabetic drugs, asthma, bibliometric analysis, insulin resistance, metabolic inflammation, obesity

## Abstract

**Background:**

The association among metabolic dysfunction, antidiabetic drugs, and asthma has attracted increasing attention in recent years. However, the knowledge structure, dominant metabolic mechanisms, drug-related research hotspots, and clinical translational evidence in this interdisciplinary field remain insufficiently characterized.

**Methods:**

This study conducted a bibliometric evidence-mapping and visualization analysis of research linking metabolic dysfunction, antidiabetic drugs, and asthma published between 2006 and 2025. Data were obtained from the Web of Science Core Collection, Scopus, and PubMed. Bibliometric and visualization methods were used to identify global research trends, major knowledge domains, thematic evolution, and emerging translational frontiers.

**Results:**

A total of 1, 502 records from the Web of Science Core Collection, 6, 710 records from Scopus, and 38 PubMed-indexed clinical studies were included. Publication output showed an overall upward trend, with a marked increase in research attention after 2015. Keyword and clustering analyses indicated that obesity, insulin resistance, metabolic syndrome, inflammation, oxidative stress, adipokines, lung function, and airway hyperresponsiveness formed the dominant knowledge base of this field. In contrast, drug-related topics, including metformin, peroxisome proliferator-activated receptor γ agonists, glucagon-like peptide-1 receptor agonists, sodium–glucose cotransporter 2 inhibitors, and dipeptidyl peptidase-4 inhibitors, gradually entered the research network but generally showed relatively limited centrality and uneven clinical evidence. Reference co-citation and burst analyses further suggested that recent research frontiers are shifting toward drug-related mechanisms, asthma exacerbations, and clinical outcome evaluation.

**Conclusion:**

Research in this field has evolved from early studies of metabolic comorbidity and inflammatory mechanisms toward drug-related mechanisms, asthma outcomes, and translational evidence. The dominant knowledge base is centered on obesity, insulin resistance, metabolic inflammation, oxidative stress, and airway dysfunction, whereas drug-specific evidence remains emerging and uneven across drug classes. Future studies should integrate large-scale clinical data, prospective cohorts, and randomized controlled trials to clarify whether selected antidiabetic drugs may modify asthma outcomes in metabolically defined asthma phenotypes. This study provides an evidence-mapping perspective for understanding the metabolic, pharmacological, and clinical translational links among metabolic dysfunction, antidiabetic drugs, and asthma.

## 1 Introduction

Asthma is a common chronic airway disease characterized by variable respiratory symptoms, variable expiratory airflow limitation, airway hyperresponsiveness, and recurrent episodes of wheezing, cough, chest tightness, and dyspnea (1, 2). Although anti-inflammatory and bronchodilator therapies remain the cornerstone of asthma management, and biologic therapies have expanded treatment options for selected patients with severe asthma, a subset of patients still experience poor symptom control, recurrent exacerbations, and impaired quality of life (2, 3). Meanwhile, diabetes, particularly type 2 diabetes, imposes a substantial global burden as a chronic metabolic disease (4). Type 2 diabetes commonly coexists with metabolic abnormalities, including obesity, insulin resistance, dyslipidemia, metabolic syndrome, and cardiovascular disease (5, 6). Increasing evidence suggests a close relationship between asthma and type 2 diabetes, with observational and systematic-review evidence supporting their co-occurrence and potential bidirectional association (7, 8). This relationship may have clinical implications, as obesity and metabolic dysfunction have been associated with poorer asthma control, more frequent exacerbations, reduced lung function, and attenuated responses to standard asthma therapies (9–11).

Obesity, insulin resistance, and metabolic inflammation are considered important pathophysiological links between metabolic abnormalities and asthma (12, 13). Compared with traditional allergic asthma, asthma associated with obesity or metabolic dysfunction is often recognized as a heterogeneous phenotype characterized by altered lung mechanics, impaired lung function, low-grade systemic inflammation, increased airway hyperresponsiveness, and poorer treatment response (10, 11). Metabolic abnormalities may contribute to airway inflammation, airway hyperresponsiveness, and airway remodeling through multiple mechanisms, including adipokine imbalance, chronic inflammatory activation, oxidative stress, altered airway smooth muscle function, epithelial dysfunction, and dysregulated immunometabolic responses (1, 12, 14). Therefore, metabolic abnormalities may not only increase the risk of asthma but also affect asthma control, exacerbation risk, and treatment response (10, 11, 13). These findings provide a theoretical basis for further exploring whether metabolic interventions and antidiabetic drugs may influence asthma-related mechanisms and outcomes (9, 15).

Antidiabetic drugs have long been used primarily for glycemic control, but their broader cardiometabolic effects have received increasing attention in recent years (16, 17). In addition to improving glucose metabolism, several drug classes, including metformin, peroxisome proliferator-activated receptor γ (PPARγ) agonists, glucagon-like peptide-1 receptor agonists (GLP-1RAs), sodium–glucose cotransporter 2 inhibitors (SGLT2is), and dipeptidyl peptidase-4 inhibitors (DPP-4is), may improve insulin sensitivity, reduce body weight, modulate inflammatory responses, or improve cardiometabolic comorbidities (9, 16, 18). These pharmacological properties have raised interest in whether selected antidiabetic drug classes may influence asthma-related outcomes, particularly in patients with asthma complicated by obesity, type 2 diabetes, or metabolic syndrome (19–21). However, existing evidence remains fragmented, with current studies mainly focusing on selected agents, such as metformin, GLP-1RAs, and PPARγ agonists, or on specific outcomes such as asthma onset, asthma attacks, exacerbations, and obesity-related asthma phenotypes (9, 18–20). In contrast, the clinical associations, thematic evolution, and evidence base linking different antidiabetic drug classes to asthma outcomes have not been systematically characterized. Therefore, a bibliometric analysis is needed to map the knowledge structure, research hotspots, and developmental trends in this field.

Accordingly, this study systematically retrieved original articles and reviews related to metabolic dysfunction, antidiabetic drugs, and asthma from the Web of Science Core Collection and Scopus databases from 2006 to 2025, supplemented by PubMed-indexed clinical studies. Using bibliometric and visualization approaches, this study aimed to characterize publication trends, collaboration patterns, knowledge structures, and research hotspots in this interdisciplinary field, and to clarify its evolution from studies of metabolic abnormalities and inflammatory mechanisms toward drug-class-specific research, asthma outcomes, and clinical translational evidence.

## 2 Materials and methods

### 2.1 Data sources and search strategy

This study systematically searched the Web of Science Core Collection, PubMed, and Scopus databases. Taking the Web of Science Core Collection as an example, the search was conducted using the Topic field (TS), and the complete search strategy was as follows: TS=(“antidiabetic*” OR “hypoglycemic*” OR “glucose-lowering drug*” OR “antihyperglycemic*” OR “metformin*” OR “Dimethylbiguanidine” OR “Dimethylguanylguanidine” OR “Glucophage” OR “biguanide*” OR “sulfonylurea*” OR “glipizide” OR “K4024” OR “K 4024” OR “Glydiazinamide” OR “Glypidizine” OR “Glidiazinamide” OR “Minidiab” OR “Minodiab” OR “Mindiab” OR “Glucotrol” OR “Glupitel” OR “Melizide” OR “Ozidia” OR “glyburide” OR “HB420” OR “HB 420” OR “HB419” OR “HB 419” OR “Glybenclamide” OR “Glibenclamide” OR “Neogluconin” OR “Micronase” OR “Maninil” OR “Euglucon N” OR “Euglucon 5” OR “Diabeta” OR “Daonil” OR “glimepiride” OR “Amaryl” OR “Amarel” OR “Roname” OR “HOE 490” OR “thiazolidinedione*” OR “Glitazones” OR “pioglitazone” OR “AD 4833” OR “AD4833” OR “U 72107A” OR “U72107A” OR “U72, 107A” OR “Actos” OR “rosiglitazone” OR “BRL 49653” OR “BRL49653” OR “Avandia” OR “meglitinide” OR “HB 699” OR “Glycoside Hydrolase Inhibitor*” OR “alpha Glucosidase Inhibitor*” OR “Pancreatic alpha Amylase Inhibitor*” OR “Intestinal alpha Amylase Inhibitor*” OR “acarbose” OR “Precose” OR “Bay g 5421” OR “Glucobay” OR “Glucor” OR “Prandase” OR “Glumida” OR “miglitol” OR “BAY m 1099” OR “Glyset” OR “Diastabol” OR “Plumarol” OR “Dipeptidyl Peptidase IV Inhibitor*” OR “DPP 4 Inhibitor*” OR “DPP IV Inhibitor*” OR “DPP4 Inhibitor*” OR “Dipeptidyl Peptidase 4 Inhibitor*” OR “Gliptin*” OR “sitagliptin” OR “MK 0431” OR “MK0431” OR “Januvia” OR “saxagliptin” OR “BMS 477118” OR “BMS477118” OR “Onglyza” OR “linagliptin” OR “BI 1356” OR “BI1356” OR “Tradjenta” OR “Trajenta” OR “alogliptin” OR “SYR 322” OR “SYR322” OR “nesina” OR “Sodium Glucose Transporter 2 Inhibitor*” OR “SGLT 2 Inhibitor*” OR “Gliflozin*” OR “SGLT2 Inhibitor*” OR “empagliflozin” OR “BI 10773” OR “BI10773” OR “Jardiance” OR “dapagliflozin” OR “BMS 512148” OR “BMS512148” OR “Farxiga” OR “Forxiga” OR “canagliflozin” OR “Invokana” OR “ertugliflozin” OR “PF 04971729” OR “PF04971729” OR “Steglatro” OR “Glucagon Like Peptide 1 Receptor Agonist*” OR “GLP 1 Agonist*” OR “GLP 1 Receptor Agonist*” OR “GLP1 Agonist*” OR “GLP 1R Agonist*” OR “GLP1R Agonist*” OR “Incretin Mimetic*” OR “GLP 1 Analog*” OR “liraglutide” OR “NN 2211” OR “NN2211” OR “Victoza” OR “Saxenda” OR “semaglutide” OR “Ozempic” OR “Rybelsus” OR “Wegovy” OR “exenatide” OR “dulaglutide” OR “LY 2189265” OR “LY2189265” OR “Trulicity” OR “insulin” OR “Iletin” OR “Novolin”) AND TS=(“asthma*”). The search period was set from January 1, 2006 to December 31, 2025. The same search logic was applied in Scopus and PubMed. In Scopus, the search was performed using the title, abstract, and keyword fields (TITLE-ABS-KEY), whereas in PubMed, the title/abstract field ([Title/Abstract]) was used. In addition, clinical study-related publication types were incorporated into the PubMed search strategy. The detailed search strategies are provided in **Supplementary Table 1**.

It should be particularly noted that the Web of Science Core Collection, Scopus, and PubMed were included for different purposes and at different levels of analysis. Owing to its standardized citation data and comprehensive document-type indexing, the Web of Science Core Collection was used as the primary bibliometric dataset in this study for citation network analysis, keyword co-occurrence analysis, and identification of core literature. Scopus, with its broader journal coverage, was used as a supplementary validation dataset to cross-validate the robustness of the main bibliometric findings. PubMed was used as a supplementary source of clinical evidence, focusing on more refined retrieval of clinical studies.

Given the inherent differences among these databases in terms of coverage, indexing rules, metadata structure, keyword fields, and citation formats, bibliometric indicators derived from the three databases, such as publication counts, centrality, and cluster size, should not be interpreted as directly comparable numerical values. Instead, they should be understood within the analytical logic and relative trends of each individual database.

### 2.2 Literature screening and data processing

**Figure 1:**
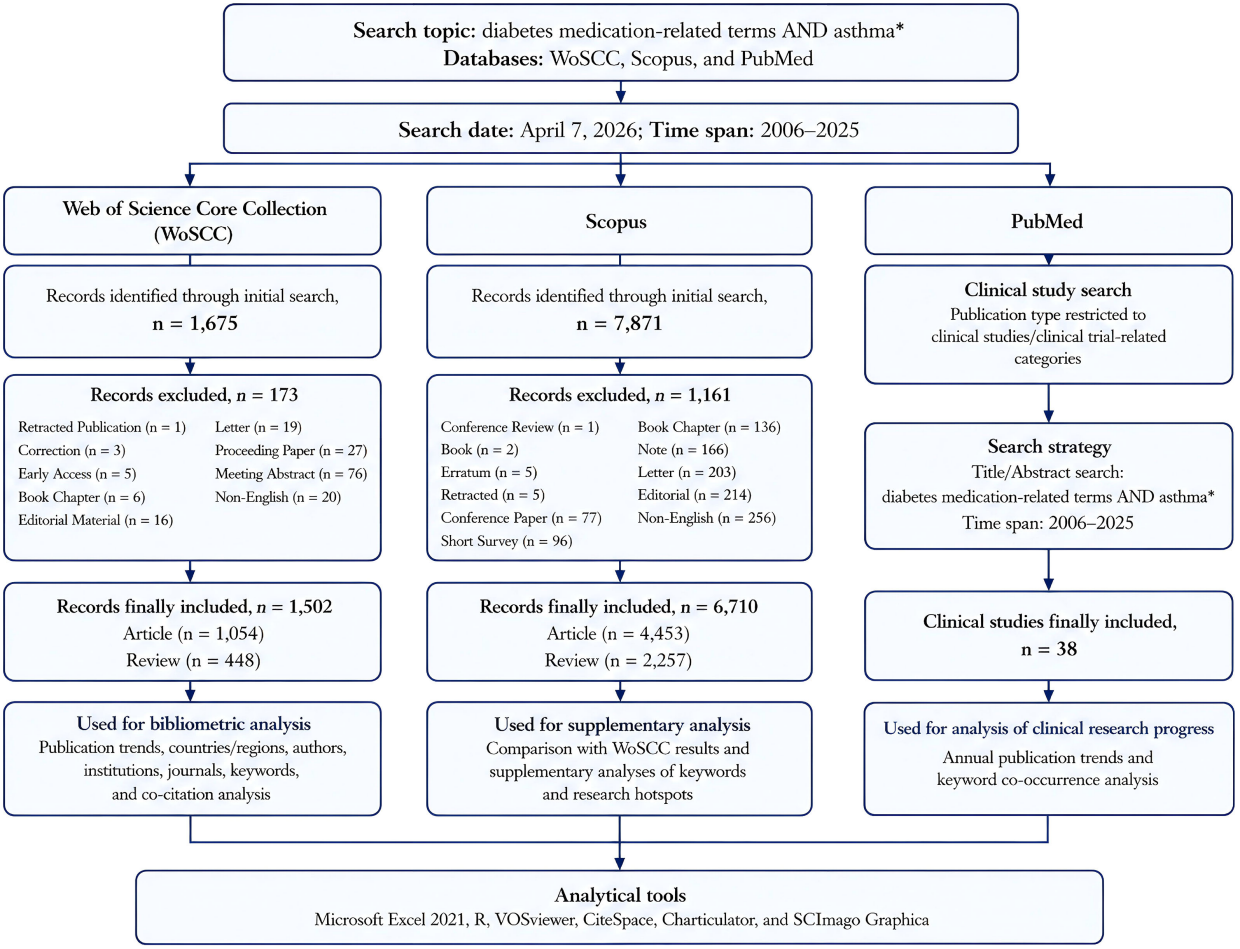
Literature screening and data processing flow. Flow diagram showing the database retrieval, deduplication, and inclusion of records from Web of Science Core Collection, Scopus, and PubMed.

In WoSCC, the initial search yielded 1, 675 records. After restricting the document types to articles and reviews and excluding non-English publications and other ineligible document types, 1, 502 records were finally included, comprising 1, 054 articles and 448 reviews. In Scopus, the initial search identified 7, 871 records. After applying the same screening criteria, 6, 710 records were included, comprising 4, 453 articles and 2, 257 reviews. The PubMed search was restricted to the title/abstract fields and publication types related to clinical studies, resulting in 38 clinical studies. The literature screening process is shown in **Figure 1**.

Bibliographic information, including titles, authors, institutions, countries/regions, journals, keywords, and references, was exported and standardized before analysis. During keyword cleaning, synonyms, abbreviations, singular and plural forms, drug class names, and overly generic terms were manually merged or removed to reduce the influence of terminological inconsistency on co-occurrence analysis and clustering results. Keyword co-occurrence analysis was then used to identify major thematic structures and research topics in this field. Terms extracted from the titles and abstracts of PubMed-indexed clinical studies were also included in subsequent co-occurrence analysis after synonym merging and removal of irrelevant terms.

### 2.3 Data analysis and visualization

The processed data were analyzed and visualized using Microsoft Excel 2021, R, VOSviewer 1.6.20, CiteSpace 6.4.R1, Scimago Graphica, and Charticulator. Descriptive statistics were performed in Microsoft Excel 2021, including basic indicators such as annual publication output, document type distribution, countries/regions, authors, institutions, and journals. The annual publication trend was assessed using the trend package in R. The Mann–Kendall trend test was used to evaluate the monotonic change in annual publication output (22), and Sen’s slope was calculated to estimate the magnitude of annual change (23).

Collaboration networks and selected thematic co-occurrence analyses were conducted using VOSviewer 1.6.20. The analyzed objects included countries/regions, authors, institutions, source journals, and terms extracted from the titles and abstracts of PubMed-indexed clinical studies. Network construction was based on the full counting method (24). Minimum inclusion thresholds were set according to the type of analysis: countries/regions with at least 10 publications were included in the country/region collaboration network; authors with at least 4 publications were included in the author collaboration network; institutions with at least 8 publications were included in the institutional collaboration network; and source journals with at least 10 publications were included in the source journal network.

CiteSpace 6.4.R1 was used to analyze keyword evolution, research frontiers, and the knowledge base of the field. The analyses included keyword co-occurrence, keyword clustering, keyword time-zone maps, keyword bursts, reference co-citation, reference clustering, reference bursts, and dual-map overlay of journals. The time span for CiteSpace analysis was set to 2006–2025, with a 1-year time slice. The node type was set as “keyword, ” “cited reference, ” or “cited journal” according to the analytical purpose. For reference co-citation analysis, the g-index was used as the selection criterion, with the parameter set to k = 25. Cluster labels were generated using the log-likelihood ratio algorithm, and modularity Q and mean silhouette S values were used to evaluate the clustering structure and consistency. Keyword and reference burst analyses were performed using the built-in burst detection function in CiteSpace to identify stage-specific research hotspots and potential frontier topics. In addition, Scimago Graphica was used to visualize annual publication trends among major contributing countries/regions, and Charticulator was used to generate a chord diagram of international collaboration. These analyses provided complementary visualization of temporal changes and global collaboration patterns in this field.

## 3 Results

### 3.1 Annual publication trends and document type distribution

**Figure 2:**
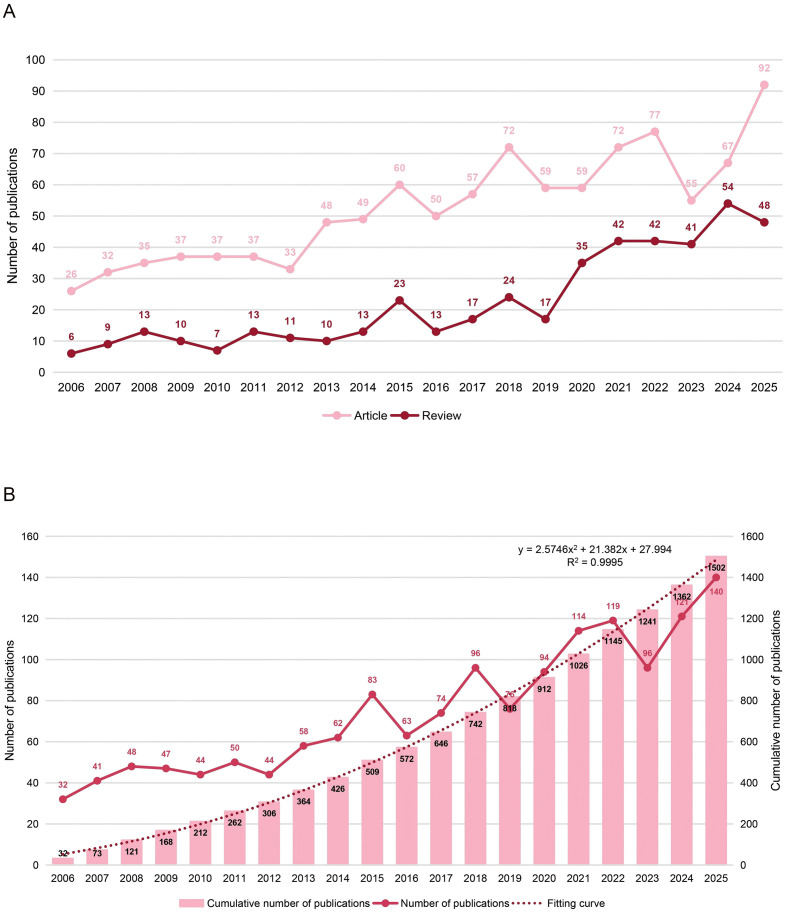
Temporal trends in publication output and document types, 2006–2025. **(A)** Annual numbers of articles and reviews. **(B)** Annual and cumulative publications; the dotted line represents the fitted trend.

As shown in **Figure 2A**, the annual numbers of both articles and reviews increased from 2006 to 2025, although their growth patterns differed. The annual number of articles increased from 26 in 2006 to 72 in 2018, followed by a fluctuating upward trend, reaching a peak of 92 in 2025. In contrast, the number of reviews remained relatively low between 2006 and 2014, with fewer than 15 reviews published in most years. The number of reviews gradually increased after 2015, with a more pronounced increase after 2020, reaching 54 and 48 in 2024 and 2025, respectively. The proportion of reviews among the two document types increased from 11%–28% in the early period to 34%–45% during 2020–2025.

From 2006 to 2025, the WoSCC dataset included 1, 054 articles and 448 reviews. Articles received 32, 401 citations in total, with an average of 30.74 citations per article, whereas reviews received 29, 101 citations in total, with an average of 64.96 citations per review. Articles accounted for approximately 70% of the included publications and contributed approximately 53% of the total citations, while reviews showed a higher average citation frequency than articles.

**Figure 2B** shows the annual publication trend in research on the association between antidiabetic drugs and asthma from 2006 to 2025. The Mann–Kendall test indicated a significant monotonic increasing trend in annual publication output (Z = 5.229, p < 0.001), with Sen’s slope suggesting an average increase of approximately 5 publications per year. Between 2006 and 2014, annual publication output remained relatively stable, with most years ranging from 40 to 62 publications. After 2015, the number of publications increased markedly, reaching 114 in 2021, 119 in 2022, 121 in 2024, and 140 in 2025. The highest annual output during the study period was observed in 2025.

### 3.2 Distribution of Web of Science categories

The 1, 502 publications in the WoSCC dataset were assigned to 106 Web of Science categories. The top 20 Web of Science categories by publication output are listed in **Supplementary Table 2**. Among them, Pharmacology & Pharmacy ranked first with 279 publications, followed by Immunology (n = 206), Respiratory System (n = 191), Biochemistry & Molecular Biology (n = 163), and Allergy (n = 152). These categories indicate that research on antidiabetic drugs and asthma mainly involves pharmacology, immunology, respiratory medicine, molecular biology, and allergy-related fields.

### 3.3 Country/region collaboration network

A total of 92 countries/regions contributed to research on antidiabetic drugs and asthma. The top ten countries in terms of publication output (NP) in this field were the United States (447 publications), China (255), India (146), the United Kingdom (86), Australia (73), South Korea (71), Italy (60), Canada (60), Germany (58), and Japan (48). Among these countries, the United States had the highest average citations per publication (AC = 57.53) and H-index (80). Italy and Germany also showed high average citation impact, with AC values exceeding 60 (**Table 1**).

**Table 1:** Top 10 countries/regions by number of publications.

Rank	Country	NP	NC	AC	H-index
1	United States	447	25714	57.53	80
2	China	255	6734	26.41	40
3	India	146	4204	28.79	33
4	United Kingdom	86	4174	48.53	33
5	Australia	73	4026	55.15	31
6	South Korea	71	2212	31.15	26
7	Italy	60	3752	62.53	27
8	Canada	60	3099	51.65	28
9	Germany	58	3945	68.02	28
10	Japan	48	2060	42.92	18

Data were obtained from the WoSCC dataset. NP, number of publications; NC, number of citations; AC, average citations per publication; H-index, Hirsch index.

**Figure 3:**
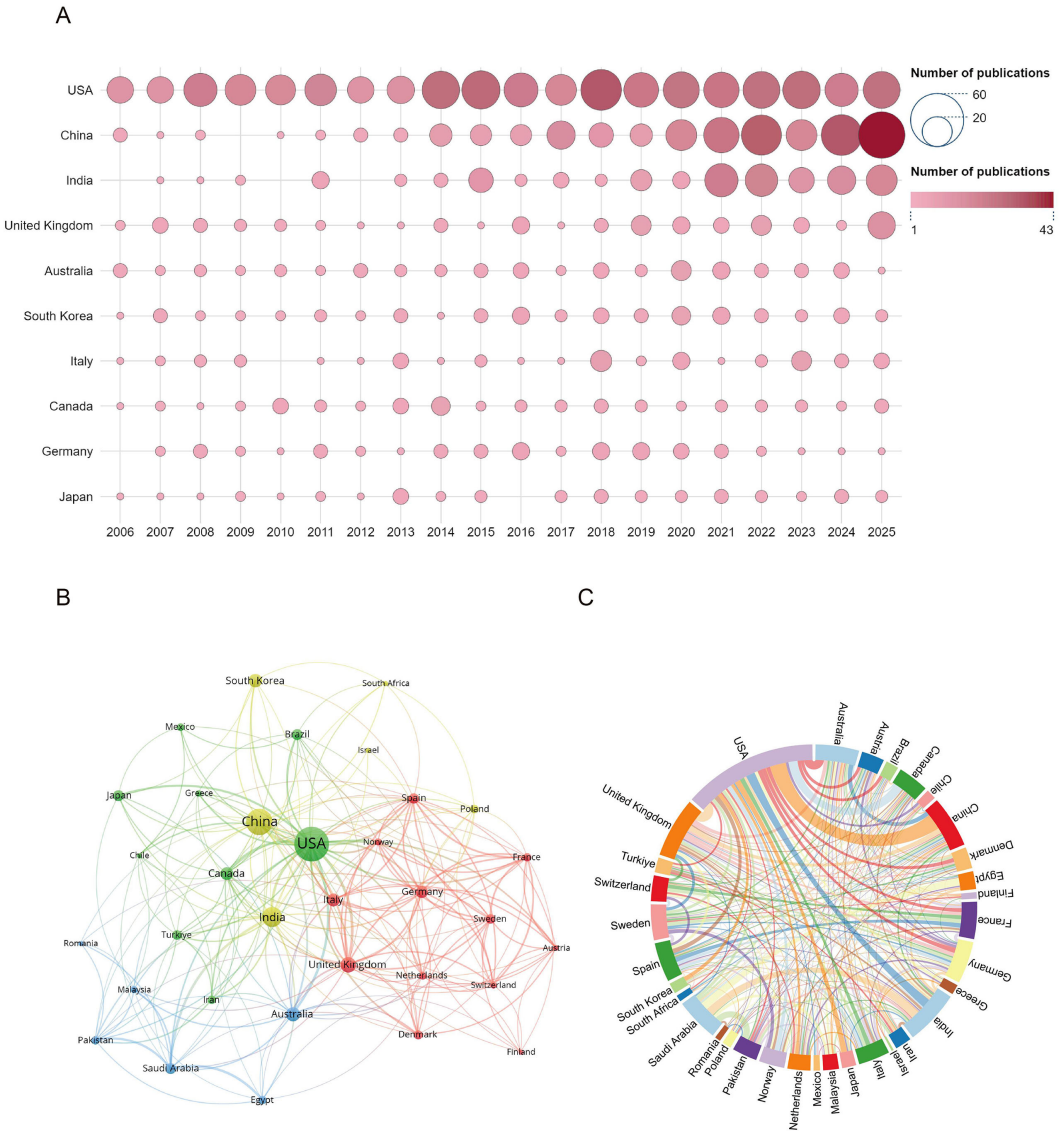
Geographic distribution and international collaboration patterns, 2006–2025. **(A)** Annual publication output of the top 10 countries/regions. **(B)** Country/region collaboration network generated by VOSviewer. **(C)** Chord diagram of major international collaborations.

**Figure 3A** shows the annual publication output of the top 10 contributing countries from 2006 to 2025. The United States maintained a leading position throughout the study period, with annual publication output fluctuating mostly between 14 and 33 publications and without a dramatic increase, indicating stable and sustained research productivity in this field. China showed the most notable growth trajectory. From 2006 to 2013, its annual output remained in single digits, with the lowest annual output being only one publication. Since 2014, China’s publication output has increased rapidly, reaching new highs in 2022 (32 publications), 2024 (33 publications), and 2025 (43 publications), and it has surpassed the United States in recent years to become the country with the highest annual publication output. India also showed a marked increase in the later period. Its publication output was limited before 2015, but increased rapidly after 2019; from 2021 to 2025, its annual publication output remained stable at 13–22 publications, making India the third most active contributor. By contrast, the United Kingdom, Australia, South Korea, Italy, Canada, Germany, and Japan showed relatively low and stable annual publication outputs, mostly fewer than 10 publications per year, without the rapid growth observed in China and India. Overall, the annual publication trends indicate increasing contributions from China and India in recent years, while the United States maintained a consistently high level of publication output throughout the study period. The country/region collaboration network included 33 countries/regions with at least 10 publications.

Country/region collaboration analysis was performed using VOSviewer, and only countries/regions with at least 10 publications were displayed, resulting in a total of 33 countries/regions. **Figure 3B** presents the visualization results. The size of each node represents publication output, and the links between nodes represent collaborations between countries/regions. To further illustrate the collaboration patterns among countries/regions and the collaborative relationships between a given country/region and others, a country/region collaboration chord diagram was generated, as shown in **Figure 3C**. The United States had a total link strength (TLS) of 188, far exceeding that of other countries/regions, and maintained strong collaborative links with China (link strength [LS] = 25), the United Kingdom (LS = 15), India (LS = 14), Canada (LS = 14), Australia (LS = 13), and Germany (LS = 7), highlighting its hub position in this field. Although China had a TLS of 72, its strongest collaboration link was with the United States (LS = 25), followed by the United Kingdom (LS = 8), Australia (LS = 7), South Korea (LS = 3), and India (LS = 3). These results indicate that China had established collaborative links with several major contributing countries/regions, although the United States remained the dominant central node in the international collaboration network. India also showed strong collaborative activity (TLS = 77), with its main collaborative links concentrated in the United States (LS = 14), Saudi Arabia (LS = 13), Egypt (LS = 4), Iran (LS = 4), and Pakistan (LS = 4), indicating close academic connections between South Asia and the Middle East. The United Kingdom (TLS = 84) collaborated frequently with the United States (LS = 15), China (LS = 8), Germany (LS = 6), Australia (LS = 6), and Italy (LS = 5), forming a stable transatlantic collaboration pattern. In addition, Saudi Arabia (TLS = 61) showed relatively strong collaboration with Egypt (LS = 12), India (LS = 13), and Pakistan (LS = 10), reflecting regional collaboration within the Middle East and South Asia. Overall, the network showed a hub-and-spoke pattern, with the United States occupying the dominant central position. China, the United Kingdom, India, Australia, and Canada showed relatively high collaboration activity and served as secondary collaborative nodes in the network.

### 3.4 Author co-authorship network

A total of 8, 670 authors contributed to research in this field. **Supplementary Table 3** lists the top 10 authors by publication output. The most productive author was Shore, Stephanie A. (16 publications; AC = 110.81; H-index = 16), followed by Forno, Erick (13 publications) and Rastogi, Deepa (13 publications). The authors ranked fourth to tenth were Holguin, Fernando; Sood, Akshay; Wood, Lisa; Wu, Tianshi David; Antunes, Edson; Donovan, Chantal; and Rehan, Virender K., with publication outputs ranging from 8 to 11 articles. Overall, the top 10 authors showed clear differences in productivity and influence, with Shore, Stephanie A. occupying a clearly leading position.

**Figure 4:**
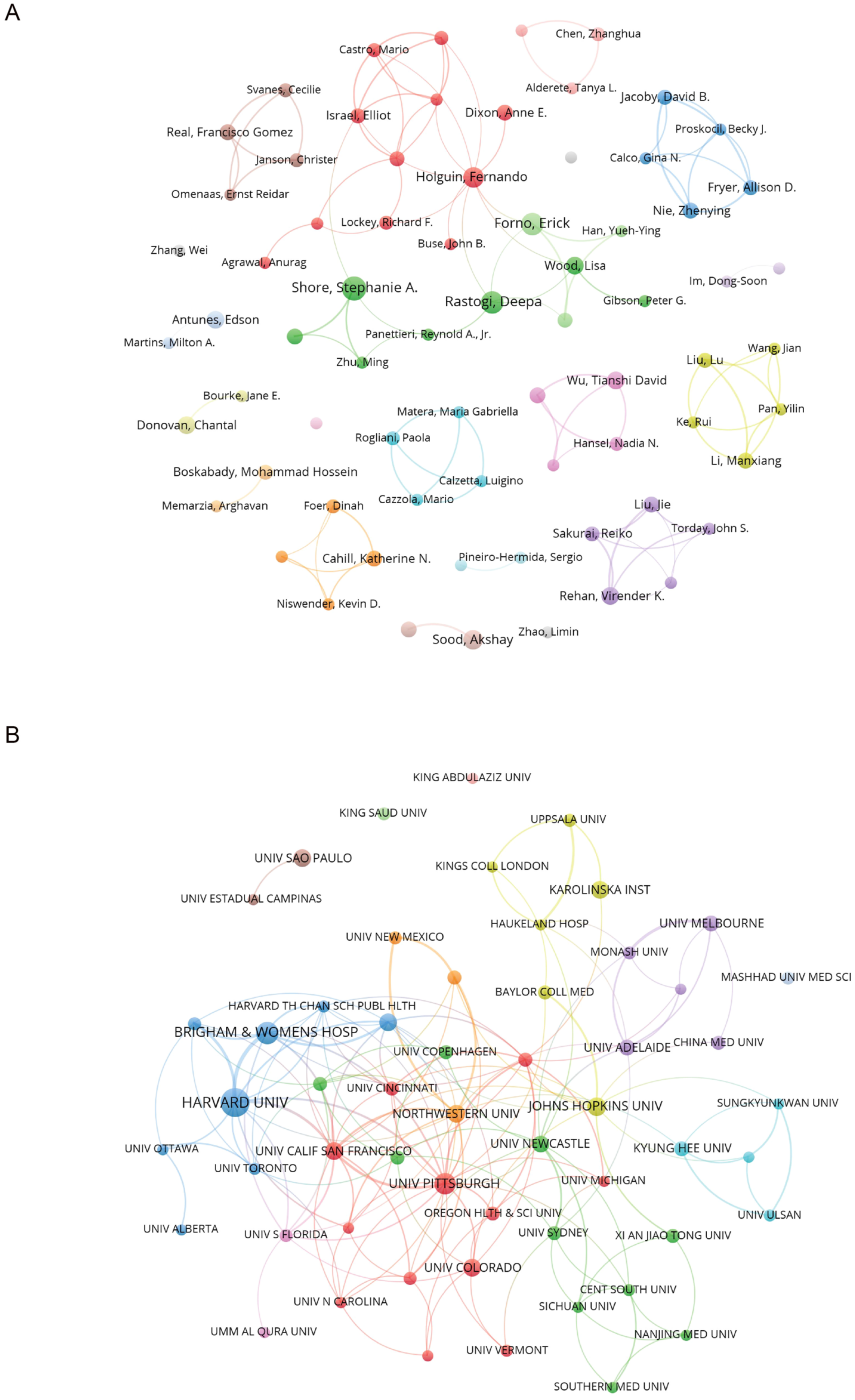
Co−authorship and institutional collaboration networks. **(A)** Co−authorship network among authors with at least 4 publications. **(B)** Collaboration network among institutions with at least 8 publications.

VOSviewer was used to analyze authors with at least 4 publications, resulting in 71 authors being included in the co-authorship network shown in **Figure 4A**. The network formed multiple relatively independent and internally connected research clusters, indicating an overall decentralized team structure. The author with the highest total link strength was Nie, Zhenying (TLS = 20), followed by Fryer, Allison D. (TLS = 18), Jacoby, David B. (TLS = 18), Li, Manxiang (TLS = 18), and Rehan, Virender K. (TLS = 18). These authors were located at the core of their respective research teams and maintained strong collaborations with authors within the same group. For example, the edge weights among Fryer, Allison D., Jacoby, David B., and Nie, Zhenying each reached 6, while the edge weight between Rehan, Virender K., and Liu, Jie reached 7. By contrast, Shore, Stephanie A., who had the highest publication output (NP = 16), had a total link strength of only 11, suggesting a relatively limited breadth of collaboration.

The main clusters in the network included Cluster 3, represented by Fryer, Allison D., Jacoby, David B., Nie, Zhenying, and Proskocil, Becky J.; Cluster 5, represented by Rehan, Virender K., Liu, Jie, Sakurai, Reiko, and Torday, John S.; Cluster 4, represented by Li, Manxiang, Liu, Lu, and Pan, Yilin; Cluster 1, represented by Holguin, Fernando, Cardet, Juan Carlos, Castro, Mario, and Israel, Elliot; Cluster 2, represented by Shore, Stephanie A., Rastogi, Deepa, and Wood, Lisa; Cluster 6, consisting mainly of Calzetta, Luigino and Cazzola, Mario; and Cluster 7, consisting mainly of Cahill, Katherine N. and Foer, Dinah. The links between clusters were sparse, and the overall network showed a multi-center, small-group structure. These findings indicate that author collaboration in this field is mainly confined to individual research teams, while extensive cross-team collaboration remains limited.

### 3.5 Institutional collaboration network

A total of 2, 454 institutions contributed to research in this field. The top 10 institutions by publication output are listed in **Supplementary Table 4**. The top ten institutions in terms of publication output were Harvard University (49 publications), Brigham and Women’s Hospital (28), the University of Pittsburgh (26), Johns Hopkins University (21), the University of California, San Francisco (19), Karolinska Institute (19), Northwestern University (18), the University of Colorado (18), Vanderbilt University (18), and the University of São Paulo (18). Among them, the University of California, San Francisco had the highest average citations per publication (AC = 91.11), whereas Harvard University had the highest H-index (32). **Figure 4B** shows the institutional collaboration network of institutions with at least 8 publications, including 55 institutions in total. Harvard University had the highest total link strength (TLS = 44), maintaining strong collaborative links with Brigham and Women’s Hospital (link strength [LS] = 13), Massachusetts General Hospital (LS = 7), and the University of California, San Francisco (UCSF; LS = 6), and occupied the central position in the network. The University of Pittsburgh had a TLS of 35 and showed close links with UCSF (LS = 6), Harvard University (LS = 4), and the University of Washington (LS = 3), forming another important hub. UCSF also had a TLS of 35 and collaborated broadly with the University of Pittsburgh, Harvard University, Northwestern University, and other institutions. Brigham and Women’s Hospital (TLS = 35), together with Harvard University, Vanderbilt University (TLS = 20), and Massachusetts General Hospital (TLS = 17), formed a dense collaborative subnetwork. In addition, institutions with relatively high TLS values included the University of Washington (TLS = 18), Monash University (TLS = 14), the University of South Florida (TLS = 14), and the University of Minnesota (TLS = 14).

In terms of clustering, the network was mainly divided into several color-coded clusters. Cluster 3 was represented by Harvard University, Brigham and Women’s Hospital, and Massachusetts General Hospital; Cluster 1 was represented by the University of Pittsburgh, UCSF, and the University of Colorado; Cluster 4 was represented by Johns Hopkins University, Karolinska Institutet, and Uppsala University; Cluster 5 was represented by the University of Adelaide, the University of Melbourne, and Monash University; and Cluster 6 was represented by Kyung Hee University, the University of Ulsan, and Sungkyunkwan University. Overall, leading institutions in the United States showed close and high-strength collaborations, forming the backbone of the network. European and Australian institutions, such as the University of Copenhagen and the University of Adelaide, had certain links with North American institutions, but institutional collaboration was still largely dominated by internal collaboration within the United States. Asian institutions, including those from China and South Korea, tended to form relatively independent subnetworks, with weaker connections to the core institutional clusters.

### 3.6 Journal analysis

**Table 2** lists the top 10 source journals by publication output and the top 10 cited journals by citation frequency. The source journals with the highest publication output in this field included the Journal of Ethnopharmacology (37 publications; IF = 5.4), American Journal of Physiology-Lung Cellular and Molecular Physiology (26 publications; IF = 3.5), and PLOS ONE (23 publications; IF = 2.6). In contrast, the most frequently cited journals were the Journal of Allergy and Clinical Immunology (2, 320 citations; IF = 11.2), American Journal of Respiratory and Critical Care Medicine (2, 152 citations; IF = 19.4), and Journal of Ethnopharmacology (1, 224 citations; IF = 5.4).

**Table 2:** Top 10 source journals and top 10 cited journals.

Rank	Journals	NP	Country	IF (JCR 2024)	Cited journals	NC	Country	IF (JCR2024)
1	JOURNAL OF ETHNOPHARMACOLOGY	37	Ireland	5.4	J ALLERGY CLIN IMMUN	2320	United States	11.2
2	AMERICAN JOURNAL OF PHYSIOLOGY-LUNG CELLULAR AND MOLECULAR PHYSIOLOGY	26	United States	3.5	AM J RESP CRIT CARE	2152	United States	19.4
3	PLOS ONE	23	United States	2.6	J ETHNOPHARMACOL	1224	Ireland	5.4
4	INTERNATIONAL JOURNAL OF MOLECULAR SCIENCES	23	Switzerland	4.9	EUR RESPIR J	1179	United Kingdom	21.2
5	JOURNAL OF ALLERGY AND CLINICAL IMMUNOLOGY	20	United States	11.2	J BIOL CHEM	1159	United States	3.9
6	SCIENTIFIC REPORTS	20	United Kingdom	3.9	PLOS ONE	1101	United States	2.6
7	JOURNAL OF ASTHMA	20	United States	1.3	J IMMUNOL	1076	United States	3.4
8	AMERICAN JOURNAL OF RESPIRATORY CELL AND MOLECULAR BIOLOGY	18	United States	5.3	DIABETES CARE	974	United States	16.6
9	ALLERGY	18	United Kingdom	12	THORAX	853	United Kingdom	8.3
10	FRONTIERS IN PHARMACOLOGY	18	Switzerland	4.8	NEW ENGL J MED	853	United States	78.5

Source journals are ranked by number of publications, whereas cited journals are ranked by citation frequency. NP, number of publications; NC, number of citations; IF, impact factor; JCR, Journal Citation Reports.

**Figure 5:**
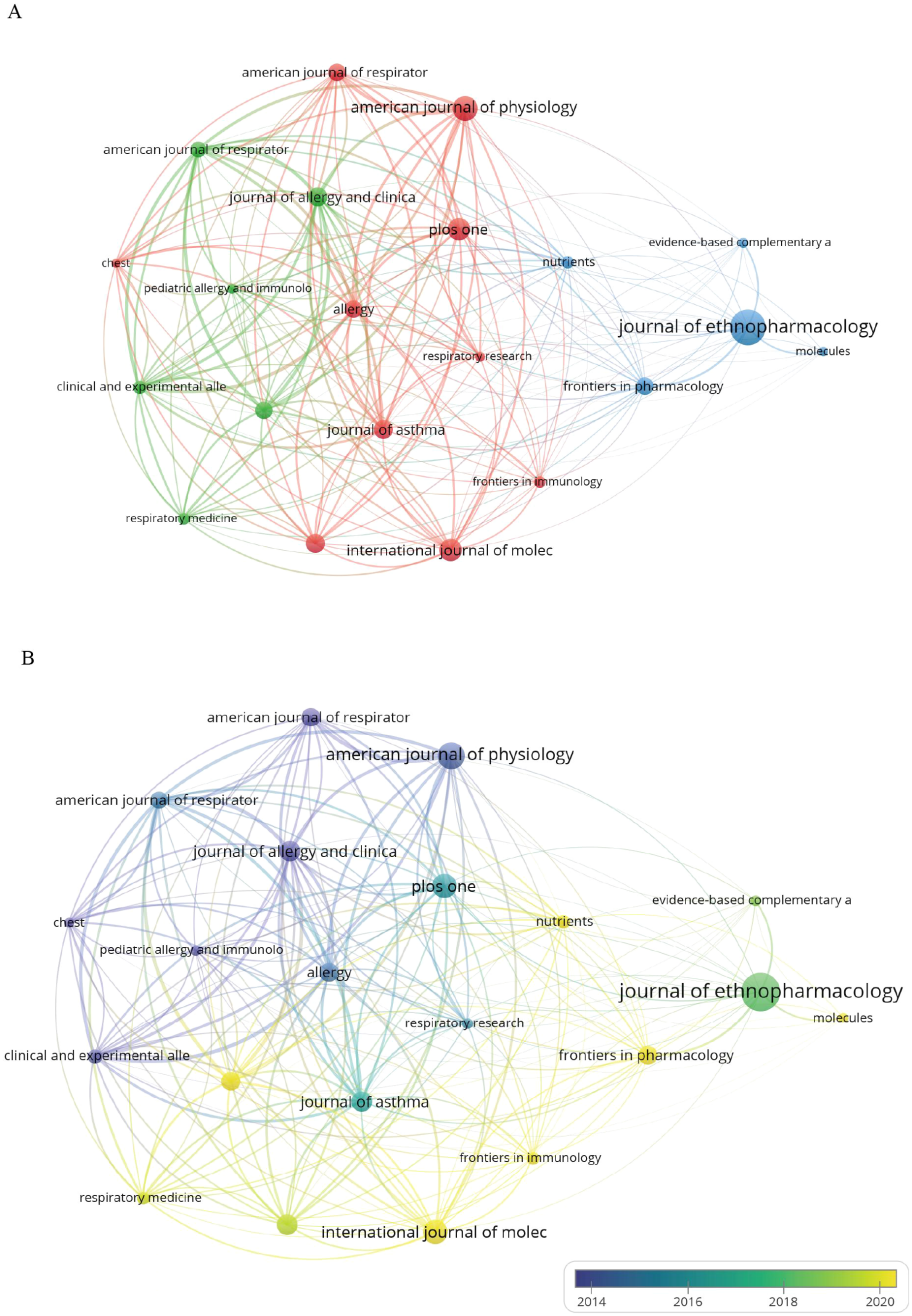
Bibliographic coupling and temporal overlay of source journals. **(A)** Bibliographic coupling network of journals with at least 10 publications. **(B)** Overlay visualization of source journals by average publication year.

**Figure 5A** shows the bibliographic coupling network of 21 source journals with at least 10 publications. Several journal groups were observed. One group mainly comprised journals in allergy, immunology, and respiratory medicine, including Journal of Allergy and Clinical Immunology, American Journal of Respiratory and Critical Care Medicine, Allergy, Clinical and Experimental Allergy, and Journal of Allergy and Clinical Immunology: In Practice. Another group was primarily composed of pharmacology and natural product research journals, such as Journal of Ethnopharmacology, Frontiers in Pharmacology, Molecules, and Evidence-Based Complementary and Alternative Medicine. A third group included journals related to basic and translational medicine, such as International Journal of Molecular Sciences, American Journal of Respiratory Cell and Molecular Biology, and American Journal of Physiology-Lung Cellular and Molecular Physiology.

**Figure 5B** presents the temporal overlay visualization of source journals based on the average publication year. Respiratory medicine and allergy/immunology journals tended to have relatively earlier average publication years, whereas journals related to pharmacology, natural products, nutrition, and molecular medicine showed relatively later average publication years.

**Supplementary Figure 1** shows the dual-map overlay between source journals and cited journals. The main citation trajectories extended from the fields of Molecular Biology, Genetics, and Health, Nursing, Medicine on the left to Molecular Biology, Immunology, and Medicine, Medical, Clinical on the right. The most prominent citation path was from Molecular, Biology, Genetics to Molecular, Biology, Immunology, with a Z-score of 7.418, suggesting that molecular biology, immunology, and clinical medicine jointly constitute the major knowledge-flow pathways in this field.

### 3.7 WoSCC keyword analysis

**Figure 6:**
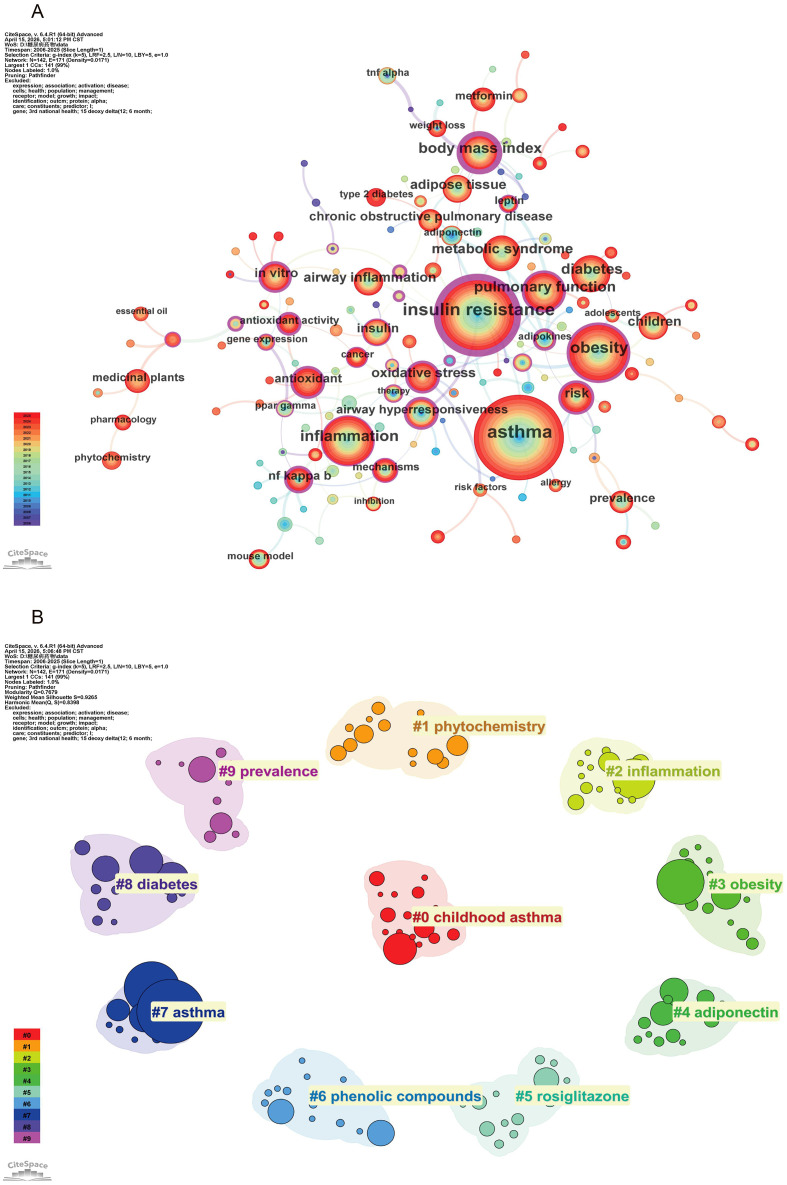
Research theme clustering and network structures of keywords in WoSCC. **(A)** Co−occurrence network of standardized keywords. **(B)** Clustering map generated using the log−likelihood ratio algorithm.

After keyword standardization, a total of 142 keywords were obtained for co-occurrence and clustering analyses. **Figure 6A** shows the keyword co-occurrence network of the WoSCC dataset. The results showed that “asthma” had the highest frequency of occurrence (628 times), but its betweenness centrality was relatively low (centrality = 0.06). By contrast, “insulin resistance” not only had a high frequency of occurrence (416 times), but also showed the highest betweenness centrality (centrality = 0.92). The “Body mass index” also showed a high centrality value (frequency = 152, centrality = 0.65). In addition, keywords such as “obesity” (frequency = 310, centrality = 0.29), “pulmonary function” (frequency = 154, centrality = 0.32), “inflammation” (frequency = 241, centrality = 0.15), “oxidative stress” (frequency = 96, centrality = 0.31), and “adipokines” (frequency = 31, centrality = 0.32) showed relatively high frequencies or high betweenness centrality, indicating that metabolic abnormalities, inflammatory responses, oxidative stress, and changes in lung function are important research themes in this field.

Compared with keywords related to metabolism and inflammation, keywords related to specific glucose-lowering drugs showed relatively lower frequencies and centrality values. “Metformin” appeared 51 times, with a centrality of 0.03; “rosiglitazone” appeared 19 times, with a centrality of 0.08; and “glucagon-like peptide-1” appeared 14 times, with a centrality of 0.08. Mechanism-related keywords, such as “NF-κB” (frequency = 59, centrality = 0.23), “PPARγ” (frequency = 28, centrality = 0.12), and “TNF-α” (frequency = 28, centrality = 0.00), also appeared in the co-occurrence network. Overall, the keyword co-occurrence results showed that research in this field is still mainly centered on obesity, insulin resistance, inflammation, oxidative stress, and airway dysfunction, whereas studies focusing on specific glucose-lowering drugs and their asthma-related outcomes have not yet formed high-centrality knowledge nodes.

Based on the keyword co-occurrence network, clustering analysis was further performed using the log-likelihood ratio algorithm in CiteSpace, as shown in **Figure 6B**. A total of 10 major clusters were generated. The modularity Q value was 0.7679, and the mean silhouette S value was 0.9265, indicating that the network had a good clustering structure and strong internal consistency. **Table 3** summarizes the keyword clusters, representative keywords, and interpreted research themes. These 10 clusters can be grouped into four major thematic categories.

**Table 3:** Keyword clusters and interpreted research themes in the WoSCC dataset.

Cluster	CiteSpace label	Representative keywords	Interpreted research theme
#0	childhood asthma	body mass index, metformin, asthma control, overweight, Mendelian randomization	Body mass index, metformin, and asthma phenotype research
#1	phytochemistry	antioxidant activity, medicinal plants, extract, antidiabetic, chemical composition	Antioxidant and antidiabetic activities of medicinal plants and natural products
#2	inflammation	NF-κB, TNF-α, allergic inflammation, mast cells, mouse model	Inflammatory signaling pathways and airway immune mechanisms
#3	obesity	obesity, childhood obesity, incident asthma, GLP-1, metabolomics	Obesity, childhood asthma, and novel metabolic targets
#4	adiponectin	adipokines, adiponectin, leptin, macrophages, T cells	Adipokine-mediated metabolic-immune interactions
#5	rosiglitazone	insulin, PPARγ, rosiglitazone, airway smooth muscle, epithelial cells	Insulin signaling, PPARγ agonists, and airway cellular responses
#6	phenolic compounds	phenolic compounds, flavonoids, molecular docking, in vitro, anti-inflammatory	Pharmacological screening of natural products and in vitro mechanistic studies
#7	asthma	insulin resistance, metabolic syndrome, airway hyperresponsiveness, airway remodeling	Asthma associated with insulin resistance and metabolic syndrome
#8	diabetes	diabetes, oxidative stress, pulmonary function, metformin use, systemic inflammation	Diabetes, oxidative stress, and pulmonary function impairment
#9	prevalence	risk, prevalence, cohort, cardiovascular disease, adherence	Comorbidity risk and epidemiological studies in patients with asthma

Clusters were generated in CiteSpace using the log-likelihood ratio algorithm. Interpreted themes were summarized from representative keywords.

The first category was related to metabolic dysfunction and asthma phenotypes, including Cluster #0 “childhood asthma, ” Cluster #3 “obesity, ” Cluster #7 “asthma, ” and Cluster #8 “diabetes.” These clusters mainly involved keywords such as “body mass index, ” “obesity, ” “insulin resistance, ” “metabolic syndrome, ” “diabetes, ” “pulmonary function, ” and “airway hyperresponsiveness.” The second category was related to metabolic inflammation and immune mechanisms, including Cluster #2 “inflammation” and Cluster #4 “adiponectin.” These clusters mainly involved “NF-κB, ” “TNF-α, ” “oxidative stress, ” “adipokines, ” “adiponectin, ” “leptin, ” “macrophages, ” and “T cells.” The third category was related to drug and pharmacological research, including Cluster #1 “phytochemistry, ” Cluster #5 “rosiglitazone, ” and Cluster #6 “phenolic compounds.” These clusters mainly involved keywords such as “metformin, ” “PPARγ, ” “rosiglitazone, ” “medicinal plants, ” “flavonoids, ” “molecular docking, ” and “anti-inflammatory.” The fourth category was related to epidemiology and comorbidity risk, mainly corresponding to Cluster #9 “prevalence, ” and involved keywords such as “risk, ” “prevalence, ” “cohort, ” “cardiovascular disease, ” and “adherence.” Among these themes, insulin resistance, obesity, and body mass index showed high centrality in the co-occurrence network, suggesting their important roles in connecting metabolic abnormalities with asthma-related research themes.

**Figure 7:**
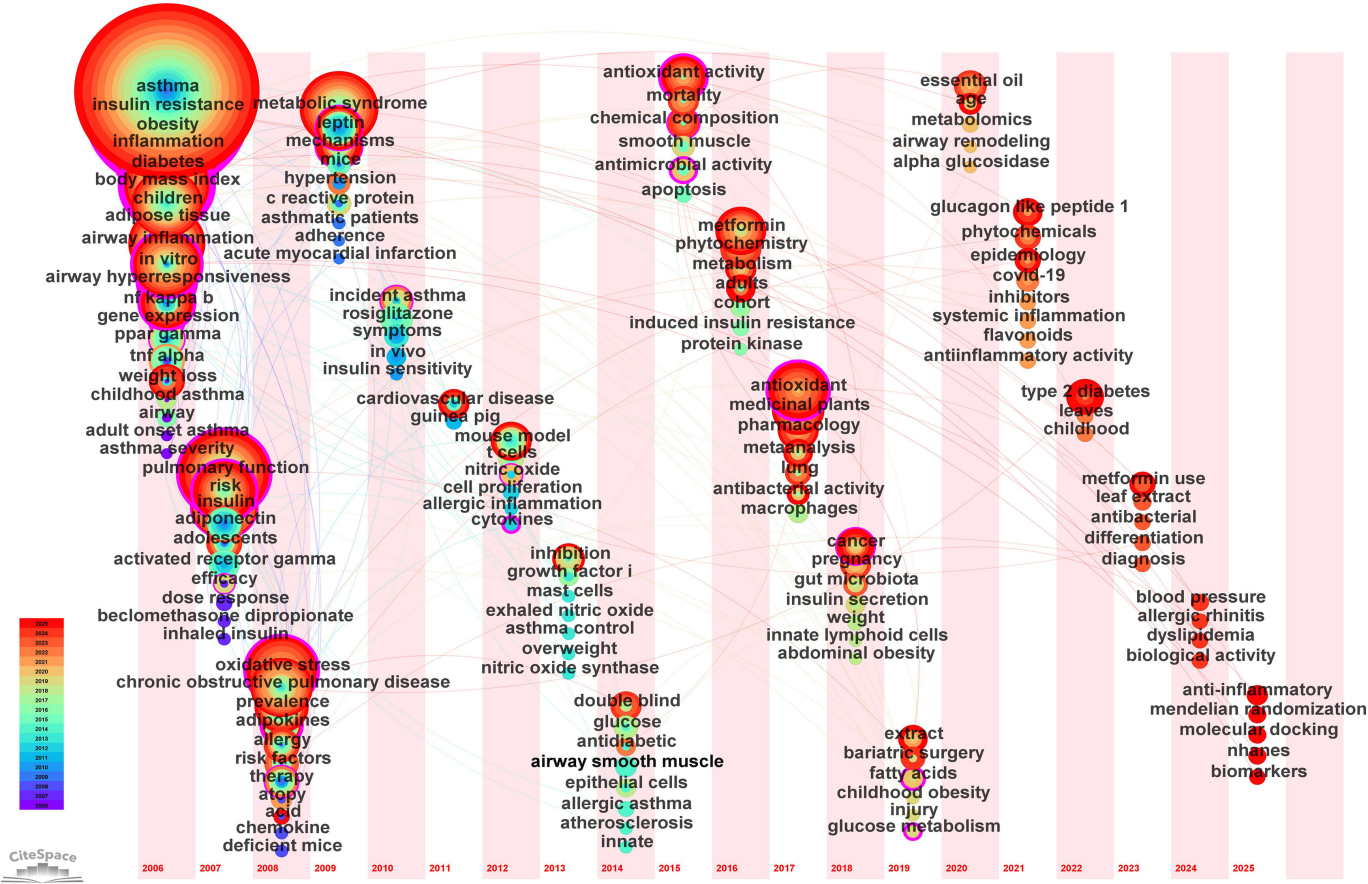
Temporal evolution of research keywords, 2006–2025. The keyword time−zone map based on the WoSCC dataset, showing the year of first appearance for major terms.

The keyword time-zone map in **Figure 7** shows the temporal distribution of keywords, with the x-axis representing the time at which each keyword first appeared. Based on the timing of keyword emergence and thematic evolution, research in this field can be roughly divided into four stages.

#### 3.7.1 Mechanistic foundation (2006–2010)

During this period, core keywords such as “asthma, ” “insulin resistance, ” “obesity, ” “body mass index, ” “inflammation, ” “NF-κB, ” “PPARγ, ” “TNF-α, ” “adipose tissue, ” “adiponectin, ” “leptin, ” and “metabolic syndrome” appeared intensively, indicating that early studies mainly focused on the relationships among obesity, insulin resistance, metabolic syndrome, and airway inflammation. At the same time, the emergence of keywords such as “rosiglitazone, ” “insulin sensitivity, ” and “incident asthma” suggested that PPARγ agonists and the risk of new-onset asthma had begun to enter the research field. This stage initially established a basic knowledge framework centered on the relationships among metabolic abnormalities, inflammatory responses, and airway dysfunction.

#### 3.7.2 Metabolic inflammation expansion (2011–2015)

Keywords gradually expanded from broad metabolic abnormalities to cellular and molecular mechanisms, including “cytokines, ” “T cells, ” “nitric oxide, ” “allergic inflammation, ” “mouse model, ” “mast cells, ” “epithelial cells, ” “airway smooth muscle, ” “apoptosis, ” and “antioxidant activity.” Related studies began to focus on the roles of immune cells, airway epithelium, airway smooth muscle, oxidative stress, and apoptosis in metabolism-related asthma. Meanwhile, the emergence of terms such as “double-blind, ” “asthma control, ” “mortality, ” and “quality of life” suggested that clinical study design and patient-related outcomes were receiving increasing attention.

#### 3.7.3 Phenotype and drug-mechanism emergence (2016–2020)

Keywords such as “metformin, ” “cohort, ” “meta-analysis, ” “gut microbiota, ” “pregnancy, ” “innate lymphoid cells, ” “abdominal obesity, ” “glucose metabolism, ” “airway remodeling, ” and “metabolomics” appeared successively. These terms indicate that the research paradigm gradually expanded from traditional mechanistic exploration to cohort studies, evidence synthesis, microbiota research, multi-omics analysis, and studies of specific populations. Metformin began to emerge as a more clearly defined research topic among specific antidiabetic drugs, whereas gut microbiota, metabolomics, and airway remodeling suggested that the field was moving toward more complex immunometabolic mechanisms.

#### 3.7.4 Clinical translation and outcome evaluation (2021–2025)

In recent years, keywords such as “glucagon-like peptide-1, ” “type 2 diabetes, ” “metformin use, ” “COVID-19, ” “systemic inflammation,” “Mendelian randomization, ” “biomarkers, ” “molecular docking, ” and “NHANES” have emerged as new themes. These keywords indicate that research attention has further shifted toward novel metabolic drugs, public population databases, causal inference, biomarkers, and computational pharmacology. The emergence of GLP-1RA-related topics and “metformin use” suggests that the association between glucose-lowering drug exposure and asthma outcomes is attracting increasing attention. However, at this stage, these topics mainly represent research hotspots and potential directions and should not be directly interpreted as mature clinical evidence.

**Supplementary Figure 2** shows that keyword burst analysis further complemented the thematic evolution revealed by the time-zone map. Early burst keywords were mainly concentrated on metabolic inflammation and nuclear receptor signaling pathways, including “adiponectin, ” “leptin, ” “adipokines, ” “PPARγ, ” and “activated receptor gamma, ” suggesting the important role of adipokines and PPARγ-related mechanisms in linking obesity, metabolic abnormalities, and asthma. Subsequently, the bursts of keywords such as “mouse model, ” “rosiglitazone, ” and “prevalence” reflected a gradual expansion of research from basic mechanisms to animal models, specific drugs, and epidemiological investigations. After 2016, keywords including “pregnancy, ” “inhibition, ” “extract, ” “medicinal plants, ” and “essential oil” emerged successively as burst terms, indicating increasing attention to specific populations, inhibitory mechanisms, and natural product pharmacology.

In recent years, keywords with relatively high burst strength and persistence until 2025 included “antioxidant, ” “phytochemistry, ” “type 2 diabetes, ” “chemical composition, ” “oxidative stress, ” and “metformin use, ” suggesting that current hotspots are concentrated on oxidative stress, phytochemistry, type 2 diabetes comorbidity, and the clinical use of metformin. Meanwhile, the emergence of keywords such as “Mendelian randomization, ” “NHANES, ” “biomarkers, ” and “molecular docking” indicates that causal inference, large-scale population databases, biomarkers, and computational pharmacology methods are gradually being introduced into this field.

### 3.8 Keyword validation and supplementary analysis based on the Scopus database

A total of 6, 710 records were retrieved from the Scopus database. During CiteSpace data conversion, 14 records with missing major information were automatically excluded, and 6, 696 records were finally included for keyword analysis. After keyword extraction, synonym merging, and removal of irrelevant terms, 128 standardized keywords were obtained for co-occurrence and clustering analyses.

**Supplementary Figure 3A** and **S3B** show the keyword co-occurrence network and clustering results based on the Scopus database, respectively. The co-occurrence network showed that “asthma” (frequency = 5, 722, centrality = 0.98) and “humans” (frequency = 6, 159, centrality = 1.30) were dominant nodes. Metabolism-related terms, including “type 2 diabetes mellitus” (frequency = 2, 905, centrality = 0.29), “obesity” (frequency = 1, 639, centrality = 0.28), “hypertension” (frequency = 2, 218, centrality = 0.48), and “inflammation” (frequency = 892, centrality = 0.14), also showed high frequencies or relatively high centrality values. The clustering analysis generated 12 clusters, with a modularity Q value of 0.8322 and a mean silhouette S value of 0.9607, indicating a clear clustering structure and high internal consistency. Together, these results further supported the “metabolism–inflammation–airway” axis as a major knowledge structure in this field.

Consistent with the WoSCC results, specific glucose-lowering drugs, including “metformin” (frequency = 572, centrality = 0.00), “rosiglitazone” (frequency = 13, centrality = 0.00), “DPP-4 inhibitor” (frequency = 81, centrality = 0.00), “GLP-1 receptor agonist” (frequency = 36, centrality = 0.00), and “SGLT2 inhibitor” (frequency = 32, centrality = 0.03), were present in the Scopus network but generally showed very low or zero centrality. Notably, the Scopus analysis supplemented several clinical and pharmacological dimensions that were less prominent in the WoSCC results. These included anti-asthmatic drugs such as “corticosteroid” and “β2-agonist, ” clinical outcome-related terms such as “comorbidity, ” “hospitalization, ” “disease severity, ” “adverse events, ” and “drug safety, ” as well as clusters related to newer glucose-lowering drugs, randomized controlled trial-related terms, cardiovascular comorbidities, allergic and atopic diseases, and COVID-19-related respiratory manifestations. The appearance of pandemic-related keywords, including “COVID-19, ” “SARS-CoV-2, ” and “pandemic, ” and psychiatric comorbidity terms such as “depression” and “anxiety, ” further expanded the emerging clinical contexts that were not fully reflected in the WoSCC results.

Overall, the Scopus analysis not only validated the major metabolic and inflammatory themes identified in the WoSCC dataset, but also provided broader clinical and pharmacological context. These findings suggest that research in this field has increasingly incorporated real-world patient outcomes, multimorbidity management, drug safety, and potential outcome associations of newer glucose-lowering drugs. Therefore, the Scopus results served as a supplementary validation of the WoSCC findings rather than as directly comparable bibliometric evidence.

### 3.9 Reference co-citation and burst analyses

Based on the g-index, with the parameter set to k = 25, references from the 1, 502 included publications were extracted. A total of 937 cited references were identified and subjected to co-citation and clustering analyses to explore the core knowledge base and the evolution of research hotspots in this field.

**Figure 8:**
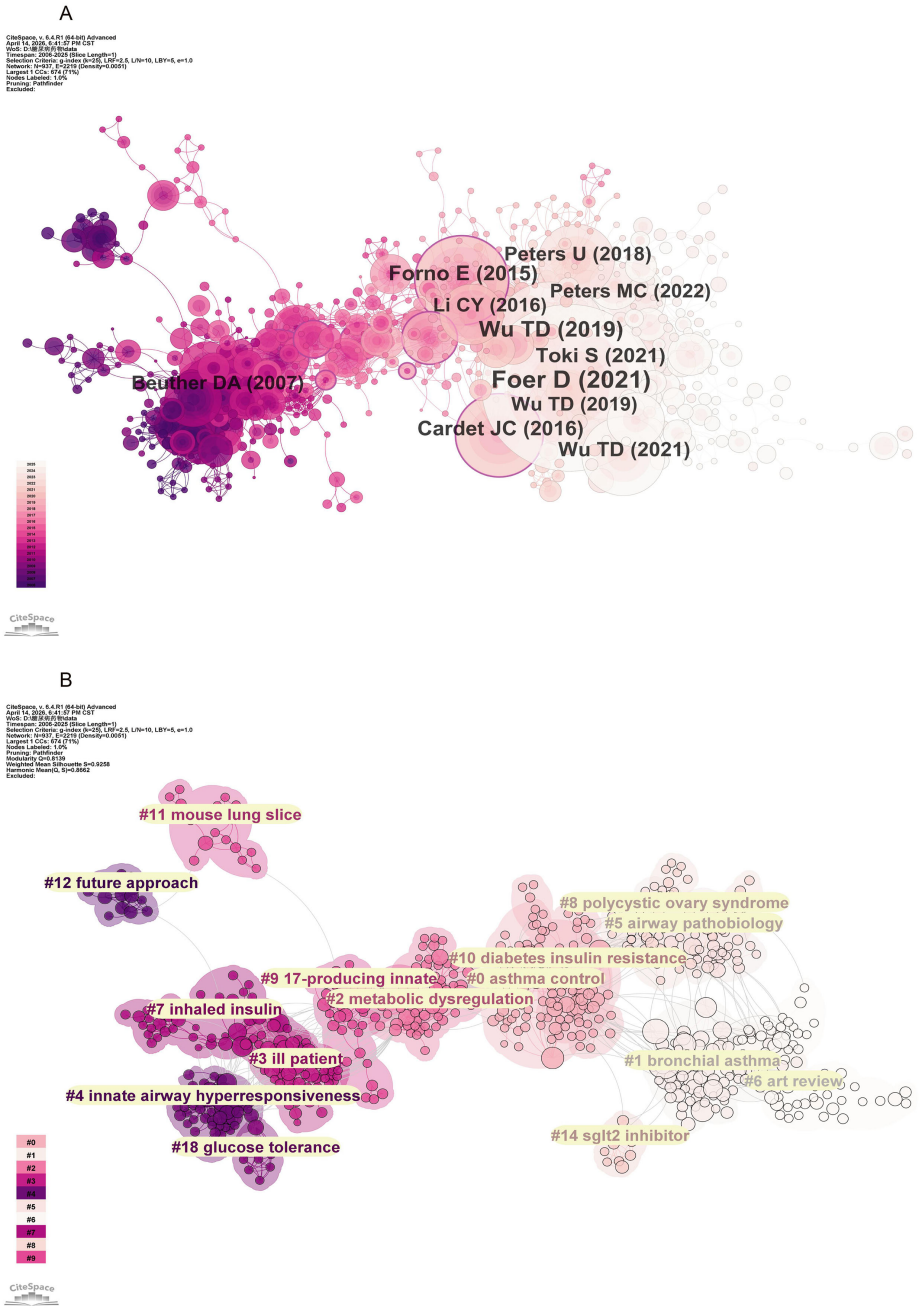
Co−citation network and thematic clustering of cited references. **(A)** Reference co−citation network based on the WoSCC dataset. **(B)** Clustering map generated using the log−likelihood ratio algorithm.

The reference co-citation analysis identified 937 cited references. As shown in **Figure 8A**, the study published by Foer et al. in the American Journal of Respiratory and Critical Care Medicine in 2021 had the highest co-citation frequency (n = 55). This reference also showed the strongest citation burst (strength = 19.15, 2022–2025). **Figure 8B** shows the results of reference clustering analysis, which generated 15 major clusters. The modularity Q value was 0.8139, and the mean silhouette S value was 0.9258, indicating a relatively clear clustering structure. The main clusters included Cluster #0 “asthma control, ” Cluster #1 “bronchial asthma, ” Cluster #2 “metabolic dysregulation, ” Cluster #4 “innate airway hyperresponsiveness, ” Cluster #5 “airway pathobiology, ” Cluster #10 “diabetes insulin resistance, ” and Cluster #14 “SGLT2 inhibitor.”

**Supplementary Figure 4** shows the cited references with the strongest citation bursts. Early burst references mainly focused on obesity, insulin resistance, and asthma risk, whereas recent burst references were more closely related to clinical outcome topics, including GLP-1 receptor agonists, metformin use, type 2 diabetes, and asthma exacerbations.

### 3.10 Clinical progress analysis based on PubMed data

**Figure 9:**
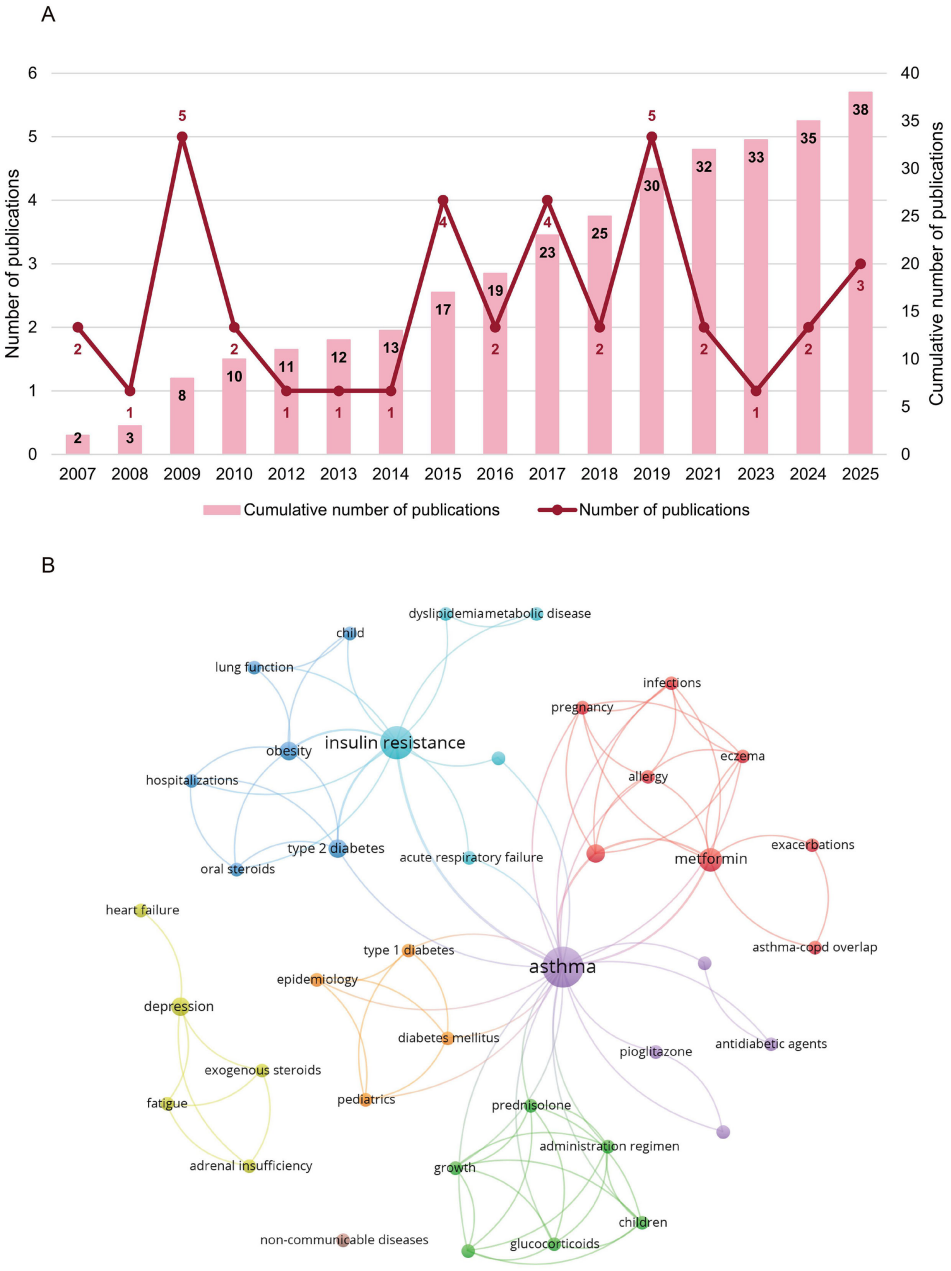
Clinical research trends and thematic network from PubMed−indexed studies. **(A)** Annual and cumulative number of clinical studies, 2007–2025. **(B)** Co−occurrence network of keywords extracted from clinical studies, generated by VOSviewer.

**Figure 9A** shows the annual publication output of PubMed-indexed clinical studies. Clinical research in this field began in 2007 and remained sparse and discontinuous throughout the study period. Although several small increases were observed in certain years, the annual number of clinical studies generally remained low, with no eligible publications identified in 2011, 2020, or 2022. By 2025, only 38 PubMed-indexed clinical studies had been identified. Compared with the much larger body of basic, mechanistic, and review literature in the WoSCC and Scopus datasets, the limited and temporally scattered clinical evidence suggests that the clinical evidence base in this interdisciplinary field remains at an early exploratory stage.

To improve the transparency and clinical interpretability of the PubMed-based evidence mapping, the detailed characteristics of the 38 PubMed-indexed clinical studies are summarized in **Supplementary Table 5**. This table includes clinical category, study design, population, sample size, drug exposure or clinical factor, comparator, asthma-related outcomes or assessed outcomes, follow-up duration, adjustment factors or statistical handling, and main findings. These studies covered heterogeneous clinical contexts, including direct antidiabetic drug exposure and asthma-related outcomes, PPARγ agonist or glucose-lowering drug intervention studies, insulin or pulmonary drug-delivery safety studies, metabolic dysfunction-related asthma outcomes, corticosteroid-related metabolic or endocrine safety studies, and other medication safety or healthcare-use studies.

Based on the 38 clinical studies retrieved from PubMed, keywords were extracted using VOSviewer. A total of 64 keywords were initially obtained. After manually merging synonyms and removing generic or irrelevant terms, 40 standardized keywords were retained for co-occurrence analysis. **Figure 9B** shows the keyword co-occurrence network generated using VOSviewer. The network was centered on “asthma” (frequency = 9, total link strength = 28) and connected several clinical dimensions, including metabolic comorbidity, glucocorticoid exposure, hospitalization, lung function, drug safety, pregnancy, polycystic ovary syndrome, and allergic comorbidities. The clustering results suggested that PubMed-indexed clinical studies were mainly organized around several themes: pediatric glucocorticoid therapy and growth-related concerns; obesity, type 2 diabetes, hospitalization, and oral steroid use; steroid-associated adverse effects; insulin resistance and metabolic comorbidities; and antidiabetic drug-related topics, particularly metformin and pioglitazone. These findings indicate that the clinical literature is still limited and heterogeneous, but it has gradually extended from glucocorticoid safety and pediatric outcomes to metabolic comorbidities, drug exposure, and selected asthma-related clinical outcomes.

Among the identified clinical studies, metformin was the most frequently investigated antidiabetic drug in the PubMed clinical dataset, and several observational studies suggested associations with improved asthma-related outcomes in selected populations (25, 26). An observational study of pediatric patients with asthma and coexisting diabetes or elevated blood glucose levels reported that metformin use was associated with a 42% reduction in systemic corticosteroid courses (25). In adults with asthma–chronic obstructive pulmonary disease overlap (ACO), metformin use was also associated with fewer total exacerbations and severe exacerbations, whereas this association was not observed in patients with COPD alone (26).

By contrast, studies on thiazolidinediones (TZDs) generally yielded negative or inconsistent findings (27–32). In patients with obesity and poorly controlled asthma, pioglitazone did not improve airway responsiveness, asthma control, or quality of life, but was associated with significant weight gain (29). In patients with mild asthma, pioglitazone also failed to improve lung function or markers of airway inflammation (28). A randomized, placebo-controlled, double-blind crossover trial of pioglitazone in severe asthma was terminated early because of lack of efficacy and clinically relevant adverse events, including foot edema and angioedema (27). Rosiglitazone produced a modest bronchodilatory effect in smokers with asthma and attenuated the late asthmatic response by approximately 15% in an allergen challenge model; however, these effects were modest and did not translate into consistent improvements in clinical asthma outcomes (31, 32). In a randomized clinical study of patients with asthma complicated by coronary heart disease, the addition of pioglitazone to standard treatment was associated with improved disease control and reduced systemic inflammatory markers, suggesting that this drug may have potential benefits in highly selected patients with specific cardiometabolic comorbidities (30).

Hyperglycemia-related evidence highlighted the adverse impact of acute metabolic dysregulation on asthma outcomes. During acute asthma exacerbations, hyperglycemia was associated with a significantly longer hospital stay, regardless of the route of insulin administration (33). Evidence from special metabolic populations also suggested that women with polycystic ovary syndrome had a higher prevalence of asthma and impaired lung function characterized by mixed obstructive and restrictive patterns, whereas metformin use during pregnancy did not appear to affect respiratory function at follow-up (34).

Taken together, these clinical studies suggest that metformin may be associated with potential respiratory benefits in selected patients with asthma or ACO, whereas TZDs have not shown consistent clinical efficacy and may raise safety concerns (25–32). Hyperglycemia-related evidence further indicates that acute metabolic dysregulation, particularly hyperglycemia, may adversely affect asthma outcomes (33). Although bibliometric findings indicate increasing research attention to glucose-lowering drugs and asthma-related outcomes, the available clinical evidence remains limited, heterogeneous, and insufficient for direct clinical translation. Therefore, the PubMed-based clinical evidence should be interpreted as structured clinical evidence mapping rather than as definitive evidence supporting drug-class-specific therapeutic effects.

## 4 Discussion

### 4.1 Overall discussion based on bibliometric findings

This study systematically mapped the knowledge structure and evolutionary trajectory of research on the association between antidiabetic drugs and asthma from 2006 to 2025 from multiple perspectives, including publication trends, country/region distribution, author and institutional collaboration, journal sources, keyword evolution, reference co-citation, and supplementary analysis of PubMed-indexed clinical studies. Overall, this field did not initially develop around the clinical question of using glucose-lowering drugs to treat asthma. Instead, it gradually emerged as an interdisciplinary research area derived from the comorbid relationships among obesity, diabetes, insulin resistance, and asthma. The annual publication output increased markedly after 2015, and the proportion of reviews has also increased in recent years, suggesting that the field has gradually moved from the accumulation of relatively scattered original studies toward a stage in which mechanistic integration, evidence synthesis, and clues for clinical translation coexist. However, the centrality of keywords related to specific glucose-lowering drugs remained low, and the number of PubMed-indexed clinical studies was limited, indicating that this field is currently closer to a mechanism-driven translational exploration stage than to a research area with mature clinical therapeutic evidence.

It should be emphasized that bibliometric indicators, including keyword co-occurrence, burst detection, cluster size, centrality, and reference co-citation, reflect research attention, frequency of occurrence, and relative position within a knowledge network. These indicators should not be interpreted as evidence of causality, therapeutic efficacy, or clinical superiority. Therefore, drug-related keywords or clusters identified in this study should be understood as emerging research hotspots or hypothesis-generating signals, rather than as direct evidence that specific antidiabetic drug classes provide established clinical benefits for asthma outcomes.

The results of country/region, author, and institutional collaboration further suggest that research influence and collaborative resources in this field remain highly concentrated. The United States has long occupied a central position in this field, reflecting its advantages in sustained research output, academic influence, and international collaboration networks. In recent years, the participation of Asian countries, such as China and India, has increased markedly, indicating a changing geographical distribution of research activity. However, the academic influence of emerging contributing countries still requires further accumulation. More importantly, the author and institutional collaboration networks showed that although several productive teams and influential institutions have been formed, the overall collaboration structure is still dominated by relatively independent small research groups, with insufficient cross-team, cross-institutional, and cross-regional collaboration. For a research topic such as antidiabetic drugs and asthma, which involves long-term drug exposure, complex comorbidity status, asthma heterogeneity, and multiple confounding factors, a fragmented collaboration pattern may limit the generation of high-quality multicenter evidence and generalizable clinical conclusions (35).

The journal distribution and dual-map overlay results indicate that this field has a clear multidisciplinary nature. Related studies are not limited to drug application itself but connect pharmacology, respiratory medicine, allergy and immunology, molecular biology, metabolic diseases, and clinical epidemiology. The major knowledge-flow pathways were concentrated among molecular biology, immunology, and clinical medicine, suggesting that research on the association between antidiabetic drugs and asthma is based on the complex interactions among metabolic abnormalities, immune inflammation, airway pathophysiology, and clinical outcomes (12). Therefore, future research should not rely solely on a single-drug or single-disease perspective. Instead, it needs to integrate basic mechanistic research, clinical phenotype identification, pharmacoepidemiology, and real-world outcome evaluation to more accurately explain the potential relationships between different antidiabetic drugs and asthma outcomes (19).

Keyword clustering, keyword burst analysis, and reference co-citation analysis jointly revealed the evolution of the knowledge base and research frontiers in this field. Early studies mainly originated from epidemiological associations among obesity, insulin resistance, metabolic syndrome, and asthma risk, and later gradually expanded to mechanisms involving inflammatory responses, oxidative stress, adipokines, PPARγ signaling, and airway hyperresponsiveness (12, 13). In recent years, topics related to metformin, GLP-1RAs, SGLT2is, DPP-4is, real-world data, and clinical outcomes have begun to enter the research frontier (9, 19). This trend suggests that research attention has gradually expanded from the comorbid association between metabolic abnormalities and asthma to whether exposure to antidiabetic drugs may influence asthma outcomes. Nevertheless, current hotspots mainly reflect changes in research attention and should not be directly interpreted as evidence of clinical efficacy. Future studies should combine large-scale real-world cohorts with more rigorous causal-inference designs, such as target trial emulation, to refine drug exposure stratification and reduce bias when randomized trials are not available (36). In addition, well-designed randomized controlled trials are still needed to clarify the actual effects of different antidiabetic drugs on asthma control, exacerbations, lung function, and safety (9).

### 4.2 Obesity, insulin resistance, and metabolic dysfunction-associated asthma

Obesity, insulin resistance, and metabolic dysfunction-associated asthma constitute one of the most important foundational research hotspots in studies on the association between antidiabetic drugs and asthma. Keyword co-occurrence analysis showed that although “asthma” was the most frequently occurring term, its centrality was not prominent. In contrast, “insulin resistance” and “body mass index” showed higher betweenness centrality, suggesting that they occupy a more important network position in connecting metabolic abnormalities, antidiabetic drugs, and asthma-related pathological processes. The high-frequency co-occurrence of keywords such as “obesity, ” “metabolic syndrome, ” “pulmonary function, ” and “airway hyperresponsiveness” further indicates that this field is gradually shifting toward the identification of asthma subphenotypes characterized by metabolic abnormalities. This finding suggests that obesity and insulin resistance may serve as key entry points for understanding the association between antidiabetic drugs and asthma.

Unlike traditional asthma research, which has mainly emphasized IgE-mediated allergic responses and local airway inflammation, metabolic dysfunction-associated asthma highlights systemic pathological links among obesity, insulin resistance, metabolic syndrome, impaired lung function, and airway hyperresponsiveness (10). Previous studies have suggested that obesity-related asthma differs from classical allergic asthma in inflammatory characteristics, patterns of lung function impairment, asthma control, and treatment response (37). This provides an important theoretical basis for the hypothesis that some antidiabetic drugs may indirectly influence asthma outcomes by improving insulin resistance, reducing body weight, or modulating metabolic inflammation (9).

Metabolic dysfunction-associated asthma may represent a candidate target population that deserves priority attention in interventional studies of glucose-lowering drugs. Patients with asthma complicated by obesity, type 2 diabetes, insulin resistance, or metabolic syndrome may differ substantially from patients with classical allergic asthma in terms of inflammatory phenotype, patterns of lung function impairment, treatment response, and exacerbation risk (38). Therefore, future studies should not use “whether a specific antidiabetic drug was used” as the only grouping variable. Instead, key covariates such as body mass index, degree of insulin resistance, diabetes status, metabolic syndrome status, and asthma phenotype should be incorporated into study design for systematic stratified analysis. Only by clearly defining these metabolic phenotypes can researchers more accurately evaluate the potential modifying effects of glucose-lowering drugs, such as metformin, GLP-1RAs, and SGLT2is, on asthma outcomes (19).

It should be noted that although keywords related to obesity, insulin resistance, and metabolic syndrome frequently appeared in the co-occurrence network, many existing studies still remain at the level of epidemiological descriptions of comorbidity associations. They are not yet sufficient to determine whether metabolic abnormalities directly modify the direction or magnitude of the effects of glucose-lowering drugs on asthma outcomes at a mechanistic level. This research gap suggests that future high-quality studies based on prospective cohorts, rigorous phenotype stratification, and mechanistic validation are needed to clarify how metabolic dysfunction-associated asthma can be used as a target phenotype for more precise patient selection and stratification (39).

### 4.3 Shared metabolic inflammatory mechanisms underlying the association between antidiabetic drugs and asthma

Chronic low-grade inflammation is a central pathological feature of obesity and type 2 diabetes mellitus (T2DM), and inflammatory activation is increasingly recognized as an important link between metabolic dysfunction and asthma (13, 40). In T2DM, immune activation, pancreatic islet inflammation, adipose tissue inflammation, and cytokine-mediated metabolic disturbance contribute to the concept of diabetes as an inflammatory disease (41). In obesity-related asthma, systemic inflammation, oxidative stress, altered adipokine signaling, and metabolic dysregulation may contribute to airway inflammation, airway hyperresponsiveness, and impaired lung function (12). Insulin resistance and obesity can promote a systemic pro-inflammatory state, leading to abnormal release of inflammatory cytokines, reactive oxygen species (ROS), and adipokines (42). These changes may interact with airway epithelial dysfunction, airway smooth muscle abnormalities, and innate and adaptive immune activation, thereby contributing to asthma heterogeneity and impaired lung function (43, 44).

The coexistence of these pathological features suggests that the potential respiratory effects of some glucose-lowering drugs are more likely to be mediated through systemic metabolic and inflammatory modulation rather than direct bronchodilatory effects. For example, metformin has been reported to attenuate airway inflammation and remodeling through AMPK-related mechanisms in experimental asthma models (45). In patients with asthma and diabetes or glycemic dysfunction, observational evidence has also suggested that metformin exposure may be associated with reduced asthma exacerbation risk, although causal inference remains limited (15). Therefore, the potential association between glucose-lowering therapy and asthma outcomes should be interpreted within a broader metabolic-inflammatory framework rather than as a direct airway-relaxing effect.

The keyword co-occurrence and clustering analyses in the bibliometric component of this study further support this interpretation. Terms such as “NF-κB, ” “TNF-α, ” “PPARγ, ” “mast cells, ” “macrophages, ” and “T cells” appeared as core nodes in thematic clusters covering both metabolic and respiratory domains. This indicates a clear shift in the field from descriptive characterization of comorbidity patterns toward mechanistic exploration of the interaction between metabolic inflammation and airway immunity. Specifically, NF-κB signaling is a central pathway linking inflammation and metabolic disease (46). In the respiratory system, NF-κB-dependent airway inflammation may also trigger systemic insulin resistance, suggesting a bidirectional inflammatory link between airway disease and metabolic dysfunction (47). In obesity-associated asthma, NLRP3 inflammasome activation and IL-17-producing innate lymphoid cells have been implicated in airway hyperreactivity, further supporting the role of innate immune-metabolic pathways in this phenotype (48).

PPARγ is the classic nuclear receptor target of thiazolidinediones (49). Existing studies suggest that PPARγ activation may participate in asthma-related pathological processes by modulating airway smooth muscle proliferation and airway remodeling (50). By contrast, the potential respiratory benefits of glucagon-like peptide-1 receptor agonists (GLP-1RAs) may be more closely related to weight reduction, improvement of metabolic status, and GLP-1 receptor-mediated anti-inflammatory effects, although their specific airway immune mechanisms require further clarification (51).

Notably, natural products, phytochemicals, and antioxidant pharmacology have formed an emerging and increasingly active branch in this research field. Oxidative stress and metabolic inflammation are mechanistically interconnected in both metabolic diseases and asthma (42, 52). Excessive ROS production can activate redox-sensitive inflammatory pathways, including NF-κB, and aggravate airway inflammation, airway hyperresponsiveness, and redox imbalance (53). Therefore, naturally derived compounds with antioxidant or anti-inflammatory activity have been proposed as candidate interventions or lead compounds for asthma-related drug development (54). However, evidence from this branch remains heterogeneous. Some studies are based on *in vitro* experiments, animal models, molecular docking, and pharmacological activity screening, whereas clinical evidence remains limited and requires further validation (55). Therefore, although natural products and phytochemicals provide mechanistic proof-of-concept and support therapeutic hypotheses, their conclusions should not be directly extrapolated to the clinical efficacy of approved glucose-lowering drugs.

Future studies should prioritize the integration of clinical biomarker data, clinical evidence, and prospective cohort designs. In particular, circulating inflammatory markers, oxidative stress indicators, adipokine profiles, and longitudinal trajectories of lung function should be systematically evaluated to determine whether they mediate the association between glucose-lowering drug exposure and asthma-related clinical outcomes. This would help move the field from mechanistic hypotheses toward rigorous clinical validation.

### 4.4 Glucose-lowering drugs as potential modifiers of asthma outcomes

Glucose-lowering drugs represent one of the research hotspots most directly related to clinical translation in studies linking metabolic dysfunction, antidiabetic drugs, and asthma. However, their emergence in the bibliometric network should be interpreted as reflecting increasing research attention rather than established clinical efficacy for asthma outcomes.

The keyword analysis in this study showed that specific drugs or drug targets, such as “metformin, ” “rosiglitazone, ” and “glucagon-like peptide-1, ” have appeared in the co-occurrence network, but their overall centrality remained relatively low. This finding indicates that current research in this field is still mainly focused on upstream mechanisms, including obesity, insulin resistance, inflammation, and oxidative stress, whereas studies directly addressing the relationship between specific glucose-lowering drugs and asthma outcomes have not yet formed a highly centralized knowledge network. This suggests that the role of glucose-lowering drugs in asthma research is still in a transitional stage from mechanistic hypothesis to clinical validation, rather than representing a therapeutic direction supported by mature evidence.

From the perspective of drug evolution, metformin and PPARγ agonists were among the classic glucose-lowering drugs that received earlier attention in this field. Thiazolidinediones have been examined in patients with diabetes and asthma, with cohort evidence suggesting a potential association with reduced asthma exacerbation risk (56). Metformin has also been evaluated in patients with diabetes and asthma, and observational studies suggest that metformin use may be associated with a lower risk of asthma exacerbation, particularly asthma-related emergency department visits or hospitalizations (57). A subsequent population-based study further examined the association between metformin use and asthma development or exacerbation in patients with type 2 diabetes (58). However, the available evidence on metformin remains mainly observational, and randomized controlled trial evidence specifically targeting asthma outcomes is still lacking (59).

The emergence of PPARγ agonists, such as rosiglitazone and pioglitazone, suggests that nuclear receptor signaling pathways may serve as potential links between metabolic abnormalities, airway smooth muscle function, and airway inflammation (60). In the keyword burst analysis, “rosiglitazone” emerged as a hotspot from 2014 to 2016, indicating that early researchers had begun to focus on the potential effects of classic glucose-lowering drugs on airway inflammation, airway smooth muscle, and asthma-related mechanisms. Experimental evidence has shown that rosiglitazone can attenuate airway inflammation and remodeling in murine asthma models (61). However, asthma-related clinical evidence for this class remains limited. In a randomized controlled trial of pioglitazone in mild asthma, 12 weeks of treatment did not improve asthma control or airway inflammation, and study recruitment was closed early partly because of safety concerns regarding pioglitazone (28). Therefore, although PPARγ agonists have mechanistic relevance, their clinical applicability in asthma remains uncertain.

In recent years, the research frontier has gradually shifted toward newer glucose-lowering drugs, including GLP-1RAs, SGLT2is, and DPP-4is. Supplementary analysis based on Scopus showed that these newer drugs have entered the keyword network, suggesting that this field is expanding from classic glucose-lowering drugs to newer therapies with broader cardiometabolic effects. Current diabetes guidelines emphasize the roles of GLP-1RAs and SGLT2is in cardiovascular and renal risk reduction in patients with type 2 diabetes (62). However, these cardiometabolic benefits should not be directly interpreted as evidence of asthma efficacy.

Among these newer agents, GLP-1RAs are of particular interest. Their potential relevance to asthma may be related to glucose lowering, weight reduction, improvement of metabolic status, and GLP-1 receptor-mediated anti-inflammatory effects (63). A review of GLP-1RAs in asthma suggested that these agents may reduce asthma exacerbations through both weight-loss-related and anti-inflammatory mechanisms, but the available clinical evidence remains limited (51). In the reference co-citation and burst analyses, the study by Foer et al. on GLP-1 receptor agonists and exacerbation risk in patients with type 2 diabetes and asthma became a core frontier reference in recent years, further suggesting that this research direction is moving from mechanistic speculation toward clinical outcome evaluation (64).

Current evidence suggests that different drug classes may differ substantially in their mechanisms of action and asthma-related associations. Metformin is mainly linked to insulin resistance, AMPK-related metabolic regulation, and anti-inflammatory effects (65). GLP-1RAs more prominently involve weight reduction, metabolic improvement, and potential immune-inflammatory modulation (66). Evidence on SGLT2is and DPP-4is in asthma remains more limited and less consistent. A recent study comparing novel antihyperglycemic drugs with metformin examined DPP-4is, GLP-1RAs, and SGLT2is in relation to asthma exacerbations, indicating that the respiratory effects of newer glucose-lowering drugs may differ by drug class (67). For SGLT2is, emerging evidence has begun to examine asthma-related outcomes in patients with type 2 diabetes, including short-term outcomes after asthma exacerbation hospitalization (68). For DPP-4is, although there is a biological rationale related to immune modulation, available clinical evidence mainly concerns asthma development or severity among patients with type 2 diabetes, and consistent evidence for respiratory benefit remains insufficient (69).

Overall, glucose-lowering drugs represent an emerging but still immature research direction in the field linking metabolic dysfunction, antidiabetic drugs, and asthma. Existing studies suggest potential associations between selected agents and asthma-related outcomes, but the evidence is still dominated by observational studies, database analyses, and preclinical mechanistic work. Therefore, these findings should be interpreted as hypothesis-generating bibliometric signals rather than as evidence supporting routine clinical use of glucose-lowering drugs for asthma treatment.

### 4.5 Clinical outcomes and drug safety

The Scopus keyword analysis showed that clinical terms such as “comorbidity, ” “hospitalization, ” “disease severity, ” “adverse events, ” “drug safety, ” “corticosteroid, ” and “β2-agonist” entered the keyword network, suggesting that this field has begun to move from mechanistic exploration toward patient-level clinical outcomes and drug safety evaluation. Based on this finding, drug safety should be a key issue in future research.

When diabetes and asthma coexist, treatment may involve concurrent exposure to glucose-lowering therapies, inhaled or systemic corticosteroids, β2-agonists, and cardiovascular or metabolic medications, which makes medication safety and drug–disease interactions clinically important (16, 69). Systemic corticosteroids can worsen glycemic control and may induce or aggravate hyperglycemia, particularly in patients with pre-existing diabetes or other metabolic risk factors (70). In addition, glucose-lowering drugs differ substantially in their effects on body weight, gastrointestinal tolerance, cardiovascular risk, infection-related outcomes, and systemic inflammatory or immune pathways; therefore, their potential influence on asthma outcomes should be evaluated within a drug-specific safety framework (16, 71). However, the PubMed-based clinical research analysis in this study showed that clinical evidence in this field remains clearly insufficient. Only 38 publications met the criteria for clinical studies, and the annual publication output remained low for a long period, with obvious temporal gaps. This indicates that current research on antidiabetic drugs and asthma is still mainly concentrated on basic mechanisms, observational associations, and literature reviews, and has not yet formed a stable body of clinical evidence.

Compared with traditional randomized controlled trials, large-scale electronic health records, insurance claims databases, prospective cohorts, and population-based databases can better capture real-world medication patterns, multimorbidity, concomitant drug use, and long-term outcomes (72, 73). In addition, existing clinical research topics remain scattered, involving children, pregnancy, obesity, type 2 diabetes, corticosteroid safety, hospitalization outcomes, lung function, and limited studies on glucose-lowering drug exposure. Systematic comparisons among specific classes of glucose-lowering drugs are still lacking.

Future studies should evaluate not only the association between specific glucose-lowering drugs and asthma exacerbations, but also drug safety, concomitant medication use, and the risk–benefit balance in patients with multimorbidity. Rigorous pharmacoepidemiologic designs are needed to minimize confounding by indication, immortal-time bias, and other time-related biases. Relevant outcomes should cover asthma control and exacerbations, lung function, medication burden, healthcare utilization, and drug-related safety events.

## 5 Limitations

This study has several limitations. Although WoSCC, Scopus, and PubMed are widely used bibliographic databases, relevant studies indexed in other databases or available only as grey literature may have been missed. Differences in database coverage, indexing rules, and document classification may also have influenced the consistency of the results. The restriction to English-language articles and reviews may have introduced language and publication bias, and may have excluded non-English publications, conference abstracts, letters, editorials, and book chapters. In addition, bibliometric results are inevitably affected by the search strategy, keyword standardization, data preprocessing, and software parameter settings. Although manual keyword cleaning was performed to reduce terminological inconsistency, some subjectivity could not be fully avoided, and some newer drug names or less frequently used terms may not have been completely captured. Moreover, bibliometric analysis can identify research hotspots, knowledge structures, and developmental trends, but it cannot establish causal relationships between antidiabetic drugs and asthma outcomes. The limited number and heterogeneity of existing clinical studies, including differences in drug exposure definitions, follow-up duration, asthma outcome measures, and confounder adjustment, further restrict direct comparisons across studies.

## 6 Conclusion

Based on data from WoSCC, Scopus, and PubMed, this study systematically mapped the bibliometric evidence, emerging trends, and translational frontiers linking metabolic dysfunction, antidiabetic drugs, and asthma from 2006 to 2025. The results showed a sustained overall increase in publication output in this field. Research topics have gradually expanded from early concerns regarding the comorbid relationships among diabetes, obesity, and asthma to metabolic inflammation, drug repurposing of glucose-lowering agents, and clinical outcome evaluation. The United States remains the dominant contributor in this field, while the participation of Asian countries, including China and India, has continued to increase.

Keyword and co-citation analyses indicated that obesity, insulin resistance, body mass index, inflammation, oxidative stress, and adipokines constitute the core knowledge base linking metabolic dysfunction, antidiabetic drugs, and asthma. Drug-related topics, including metformin, PPARγ agonists, GLP-1 receptor agonists, SGLT2 inhibitors, and DPP-4 inhibitors, have gradually emerged in the research network. However, this study should be interpreted primarily as an evidence-mapping and hypothesis-generating bibliometric analysis. Current evidence remains mainly centered on metabolic comorbidity, inflammatory mechanisms, and airway dysfunction, whereas direct clinical evidence linking specific antidiabetic drug classes to asthma outcomes remains limited. Therefore, current evidence is insufficient to support the use of antidiabetic drugs for asthma prevention or treatment outside their approved metabolic indications. Future studies should include large prospective cohorts, randomized controlled trials, and rigorous pharmacoepidemiological designs to clarify whether these emerging associations have clinical relevance and whether selected antidiabetic drug classes may modify asthma outcomes in metabolically defined asthma phenotypes.

## Data Availability

The original contributions presented in the study are included in the article/**Supplementary Material**. Further inquiries can be directed to the corresponding author.
