# Supplementary material for: Metabolic dysfunction, antidiabetic drugs, and asthma: bibliometric evidence, emerging trends, and translational frontiers from 2006 to 2025
Source: Front Endocrinol (Lausanne). 2026 Jul 22;17:1883559. doi: 10.3389/fendo.2026.1883559 (PMC13437389; doi:10.3389/fendo.2026.1883559)
Supplement: Supplementary file 1 [file SupplementaryFile1.docx]

**Supplementary Figure Legends**

**Supplementary Figure S1. Dual-map overlay of source journals and cited journals.**

**Supplementary Figure S2. Top 25 keywords with the strongest citation bursts in the WoSCC dataset, 2006–2025.**

**Supplementary Figure S3. Keyword co-occurrence and clustering analysis based on the Scopus dataset.**
**(A)** Keyword co-occurrence network generated using CiteSpace. Node size represents keyword frequency, and purple rings indicate betweenness centrality.
**(B)** Keyword clustering map generated using the log-likelihood ratio algorithm.

**Supplementary Figure S4. Top 25 cited references with the strongest citation bursts in the WoSCC dataset, 2006–2025.**
