# Supplementary material for: Metabolic dysfunction, antidiabetic drugs, and asthma: bibliometric evidence, emerging trends, and translational frontiers from 2006 to 2025
Source: Front Endocrinol (Lausanne). 2026 Jul 22;17:1883559. doi: 10.3389/fendo.2026.1883559 (PMC13437389; doi:10.3389/fendo.2026.1883559)
Supplement: Supplementary file 2 [file SupplementaryFile2.docx]

**Supplementary Table S1. Search strategies used in Scopus and PubMed.**

| **Database** | **Search field/search framework** |
| --- | --- |
| Scopus | TITLE-ABS-KEY("antidiabetic*" OR "hypoglycemic*" OR "glucose-lowering drug*" OR "antihyperglycemic*" OR "metformin*" OR "Dimethylbiguanidine" OR "Dimethylguanylguanidine" OR "Glucophage" OR "biguanide*" OR "sulfonylurea*" OR "glipizide" OR "K4024" OR "K 4024" OR "Glydiazinamide" OR "Glypidizine" OR "Glidiazinamide" OR "Minidiab" OR "Minodiab" OR "Mindiab" OR "Glucotrol" OR "Glupitel" OR "Melizide" OR "Ozidia" OR "glyburide" OR "HB420" OR "HB 420" OR "HB419" OR "HB 419" OR "Glybenclamide" OR "Glibenclamide" OR "Neogluconin" OR "Micronase" OR "Maninil" OR "Euglucon N" OR "Euglucon 5" OR "Diabeta" OR "Daonil" OR "glimepiride" OR "Amaryl" OR "Amarel" OR "Roname" OR "HOE 490" OR "thiazolidinedione*" OR "Glitazones" OR "pioglitazone" OR "AD 4833" OR "AD4833" OR "U 72107A" OR "U72107A" OR "U72,107A" OR "Actos" OR "rosiglitazone" OR "BRL 49653" OR "BRL49653" OR "Avandia" OR "meglitinide" OR "HB 699" OR "Glycoside Hydrolase Inhibitor*" OR "alpha Glucosidase Inhibitor*" OR "Pancreatic alpha Amylase Inhibitor*" OR "Intestinal alpha Amylase Inhibitor*" OR "acarbose" OR "Precose" OR "Bay g 5421" OR "Glucobay" OR "Glucor" OR "Prandase" OR "Glumida" OR "miglitol" OR "BAY m 1099" OR "Glyset" OR "Diastabol" OR "Plumarol" OR "Dipeptidyl Peptidase IV Inhibitor*" OR "DPP 4 Inhibitor*" OR "DPP IV Inhibitor*" OR "DPP4 Inhibitor*" OR "Dipeptidyl Peptidase 4 Inhibitor*" OR "Gliptin*" OR "sitagliptin" OR "MK 0431" OR "MK0431" OR "Januvia" OR "saxagliptin" OR "BMS 477118" OR "BMS477118" OR "Onglyza" OR "linagliptin" OR "BI 1356" OR "BI1356" OR "Tradjenta" OR "Trajenta" OR "alogliptin" OR "SYR 322" OR "SYR322" OR "nesina" OR "Sodium Glucose Transporter 2 Inhibitor*" OR "SGLT 2 Inhibitor*" OR "Gliflozin*" OR "SGLT2 Inhibitor*" OR "empagliflozin" OR "BI 10773" OR "BI10773" OR "Jardiance" OR "dapagliflozin" OR "BMS 512148" OR "BMS512148" OR "Farxiga" OR "Forxiga" OR "canagliflozin" OR "Invokana" OR "ertugliflozin" OR "PF 04971729" OR "PF04971729" OR "Steglatro" OR "Glucagon Like Peptide 1 Receptor Agonist*" OR "GLP 1 Agonist*" OR "GLP 1 Receptor Agonist*" OR "GLP1 Agonist*" OR "GLP 1R Agonist*" OR "GLP1R Agonist*" OR "Incretin Mimetic*" OR "GLP 1 Analog*" OR "liraglutide" OR "NN 2211" OR "NN2211" OR "Victoza" OR "Saxenda" OR "semaglutide" OR "Ozempic" OR "Rybelsus" OR "Wegovy" OR "exenatide" OR "dulaglutide" OR "LY 2189265" OR "LY2189265" OR "Trulicity" OR "insulin" OR "Iletin" OR "Novolin") AND TITLE-ABS-KEY("asthma*") |
| PubMed | (antidiabetic*[tiab] OR hypoglycemic*[tiab] OR glucose-lowering drug*[tiab] OR antihyperglycemic*[tiab] OR metformin*[tiab] OR Dimethylbiguanidine[tiab] OR Dimethylguanylguanidine[tiab] OR Glucophage[tiab] OR biguanide*[tiab] OR sulfonylurea*[tiab] OR glipizide[tiab] OR K4024[tiab] OR K 4024[tiab] OR Glydiazinamide[tiab] OR Glypidizine[tiab] OR Glidiazinamide[tiab] OR Minidiab[tiab] OR Minodiab[tiab] OR Mindiab[tiab] OR Glucotrol[tiab] OR Glupitel[tiab] OR Melizide[tiab] OR Ozidia[tiab] OR glyburide[tiab] OR HB420[tiab] OR HB 420[tiab] OR HB419[tiab] OR HB 419[tiab] OR Glybenclamide[tiab] OR Glibenclamide[tiab] OR Neogluconin[tiab] OR Micronase[tiab] OR Maninil[tiab] OR Euglucon N[tiab] OR Euglucon 5[tiab] OR Diabeta[tiab] OR Daonil[tiab] OR glimepiride[tiab] OR Amaryl[tiab] OR Amarel[tiab] OR Roname[tiab] OR HOE 490[tiab] OR thiazolidinedione*[tiab] OR Glitazones[tiab] OR pioglitazone[tiab] OR AD 4833[tiab] OR AD4833[tiab] OR U 72107A[tiab] OR U72107A[tiab] OR U72,107A[tiab] OR Actos[tiab] OR rosiglitazone[tiab] OR BRL 49653[tiab] OR BRL49653[tiab] OR Avandia[tiab] OR meglitinide[tiab] OR HB 699[tiab] OR Glycoside Hydrolase Inhibitor*[tiab] OR alpha Glucosidase Inhibitor*[tiab] OR Pancreatic alpha Amylase Inhibitor*[tiab] OR Intestinal alpha Amylase Inhibitor*[tiab] OR acarbose[tiab] OR Precose[tiab] OR Bay g 5421[tiab] OR Glucobay[tiab] OR Glucor[tiab] OR Prandase[tiab] OR Glumida[tiab] OR miglitol[tiab] OR BAY m 1099[tiab] OR Glyset[tiab] OR Diastabol[tiab] OR Plumarol[tiab] OR Dipeptidyl Peptidase IV Inhibitor*[tiab] OR DPP 4 Inhibitor*[tiab] OR DPP IV Inhibitor*[tiab] OR DPP4 Inhibitor*[tiab] OR Dipeptidyl Peptidase 4 Inhibitor*[tiab] OR Gliptin*[tiab] OR sitagliptin[tiab] OR MK 0431[tiab] OR MK0431[tiab] OR Januvia[tiab] OR saxagliptin[tiab] OR BMS 477118[tiab] OR BMS477118[tiab] OR Onglyza[tiab] OR linagliptin[tiab] OR BI 1356[tiab] OR BI1356[tiab] OR Tradjenta[tiab] OR Trajenta[tiab] OR alogliptin[tiab] OR SYR 322[tiab] OR SYR322[tiab] OR nesina[tiab] OR Sodium Glucose Transporter 2 Inhibitor*[tiab] OR SGLT 2 Inhibitor*[tiab] OR Gliflozin*[tiab] OR SGLT2 Inhibitor*[tiab] OR empagliflozin[tiab] OR BI 10773[tiab] OR BI10773[tiab] OR Jardiance[tiab] OR dapagliflozin[tiab] OR BMS 512148[tiab] OR BMS512148[tiab] OR Farxiga[tiab] OR Forxiga[tiab] OR canagliflozin[tiab] OR Invokana[tiab] OR ertugliflozin[tiab] OR PF 04971729[tiab] OR PF04971729[tiab] OR Steglatro[tiab] OR Glucagon Like Peptide 1 Receptor Agonist*[tiab] OR GLP 1 Agonist*[tiab] OR GLP 1 Receptor Agonist*[tiab] OR GLP1 Agonist*[tiab] OR GLP 1R Agonist*[tiab] OR GLP1R Agonist*[tiab] OR Incretin Mimetic*[tiab] OR GLP 1 Analog*[tiab] OR liraglutide[tiab] OR NN 2211[tiab] OR NN2211[tiab] OR Victoza[tiab] OR Saxenda[tiab] OR semaglutide[tiab] OR Ozempic[tiab] OR Rybelsus[tiab] OR Wegovy[tiab] OR exenatide[tiab] OR dulaglutide[tiab] OR LY 2189265[tiab] OR LY2189265[tiab] OR Trulicity[tiab] OR insulin[tiab] OR Iletin[tiab] OR Novolin[tiab]) AND (asthma*[tiab]) AND (Adaptive Clinical Trial[pt] OR Clinical Conference[pt] OR Clinical Study[pt] OR Clinical Trial[pt] OR Clinical Trial, Phase I[pt] OR Clinical Trial, Phase II[pt] OR Clinical Trial, Phase III[pt] OR Clinical Trial, Phase IV[pt] OR Clinical Trial Protocol[pt] OR Controlled Clinical Trial[pt] OR Pragmatic Clinical Trial[pt]) |

Note. WoSCC, Web of Science Core Collection; DPP-4, dipeptidyl peptidase-4; SGLT2, sodium-glucose cotransporter 2; GLP-1, glucagon-like peptide-1.

**Supplementary Table S2. Top 20 Web of Science categories by number of publications.**

| Rank | Web of Science Categories | Record Count | Rank | Web of Science Categories | Record Count |
| --- | --- | --- | --- | --- | --- |
| 1 | Pharmacology Pharmacy | 279 | 11 | Integrative Complementary Medicine | 67 |
| 2 | Immunology | 206 | 12 | Plant Sciences | 66 |
| 3 | Respiratory System | 191 | 13 | Pediatrics | 61 |
| 4 | Biochemistry Molecular Biology | 163 | 14 | Public Environmental Occupational Health | 59 |
| 5 | Allergy | 152 | 15 | Multidisciplinary Sciences | 56 |
| 6 | Endocrinology Metabolism | 112 | 16 | Chemistry Multidisciplinary | 55 |
| 7 | Medicine General Internal | 104 | 17 | Physiology | 51 |
| 8 | Chemistry Medicinal | 94 | 18 | Nutrition Dietetics | 44 |
| 9 | Cell Biology | 91 | 19 | Food Science Technology | 33 |
| 10 | Medicine Research Experimental | 89 | 20 | Critical Care Medicine | 31 |

*Note.* Data were obtained from the Web of Science Core Collection (WoSCC) dataset. Categories are ranked by record count.

**Supplementary Table S3. Top 10 authors by number of publications.**

| Rank | Author | NP | NC | AC | H-index |
| --- | --- | --- | --- | --- | --- |
| 1 | Shore, Stephanie A. | 16 | 1773 | 110.81 | 16 |
| 2 | Forno, Erick | 13 | 589 | 45.31 | 11 |
| 3 | Rastogi, Deepa | 13 | 540 | 41.54 | 10 |
| 4 | Holguin, Fernando | 11 | 567 | 51.55 | 10 |
| 5 | Sood, Akshay | 10 | 503 | 50.30 | 9 |
| 6 | Wood, Lisa | 8 | 599 | 74.88 | 7 |
| 7 | Wu, Tianshi David | 8 | 370 | 46.25 | 8 |
| 8 | Antunes, Edson | 8 | 277 | 34.63 | 7 |
| 9 | Donovan, Chantal | 8 | 249 | 31.13 | 8 |
| 10 | Rehan, Virender K. | 8 | 243 | 30.38 | 6 |

*Note.* Data were obtained from the WoSCC dataset. NP, number of publications; NC, number of citations; AC, average citations per publication; H-index, Hirsch index.

**Supplementary Table S4. Top 10 institutions by number of publications.**

| Rank | Organization | NP | NC | AC | H-index |
| --- | --- | --- | --- | --- | --- |
| 1 | HARVARD UNIV | 49 | 4103 | 83.73 | 32 |
| 2 | BRIGHAM & WOMENS HOSP | 28 | 1780 | 63.57 | 17 |
| 3 | UNIV PITTSBURGH | 26 | 1289 | 49.58 | 20 |
| 4 | JOHNS HOPKINS UNIV | 21 | 1610 | 76.67 | 14 |
| 5 | UNIV CALIF SAN FRANCISCO | 19 | 1731 | 91.11 | 14 |
| 6 | KAROLINSKA INST | 19 | 735 | 38.68 | 13 |
| 7 | NORTHWESTERN UNIV | 18 | 1389 | 77.17 | 14 |
| 8 | UNIV COLORADO | 18 | 1085 | 60.28 | 12 |
| 9 | VANDERBILT UNIV | 18 | 604 | 33.56 | 12 |
| 10 | UNIV SAO PAULO | 18 | 654 | 36.33 | 14 |

*Note.* Data were obtained from the WoSCC dataset. NP, number of publications; NC, number of citations; AC, average citations per publication; H-index, Hirsch index.
